# A family of N-heterocyclic carbene-stabilized borenium ions for metal-free imine hydrogenation catalysis†
†Electronic supplementary information (ESI) available: Synthetic and spectral details are deposited. CCDC 1035566–1035574. For ESI and crystallographic data in CIF or other electronic format see DOI: 10.1039/c4sc03675a


**DOI:** 10.1039/c4sc03675a

**Published:** 2015-01-26

**Authors:** Jeffrey M. Farrell, Roy T. Posaratnanathan, Douglas W. Stephan

**Affiliations:** a Department of Chemistry , University of Toronto , 80 St. George St. , Toronto , ON M5H3H6 , Canada . Email: dstephan@chem.utoronto.ca; b Department of Chemistry , Faculty of Science , King Abdulaziz University , Jeddah , Saudi Arabia

## Abstract

This manuscript probes the steric and electronic attributes that lead to “frustrated Lewis pair” (FLP)-type catalysis of imine hydrogenation by borenium ions. Hydride abstraction from (I*t*Bu)HB(C_6_F_5_)_2_**2** prompts intramolecular C–H bond activation to give (CHN)_2_(*t*Bu) (CMe_2_CH_2_)CB(C_6_F_5_)_2_**3**, defining an upper limit of Lewis acidity for FLP hydrogenation catalysis. A series of seven N-heterocyclic carbene–borane (NHC–borane) adducts ((R′CNR)_2_C)(HBC_8_H_14_) (R′ = H, R = dipp **4a**, Mes **5a**, Me **8a**; R = Me R′ = Me **9a**, Cl, **10a**) and ((HC)_2_(NMe)(NR)C)(HBC_8_H_14_) (R = *t*Bu, **6a**, Ph **7a**) are prepared and converted to corresponding borenium salts. These species are evaluated as catalysts for metal-free imine hydrogenation at room temperature. Systematic tuning of the carbene donor for the hydrogenation of archetypal substrate *N*-benzylidene-*tert*-butylamine achieves the highest reported turn-over frequencies for FLP-catalyzed hydrogenation at amongst the lowest reported catalyst loadings. The most active NHC–borenium catalyst of this series, derived from **10a**, is readily isolable, crystallographically characterized and shown to be effective in the hydrogenation catalysis of functional group-containing imines and N-heterocycles.

## Abstract 2

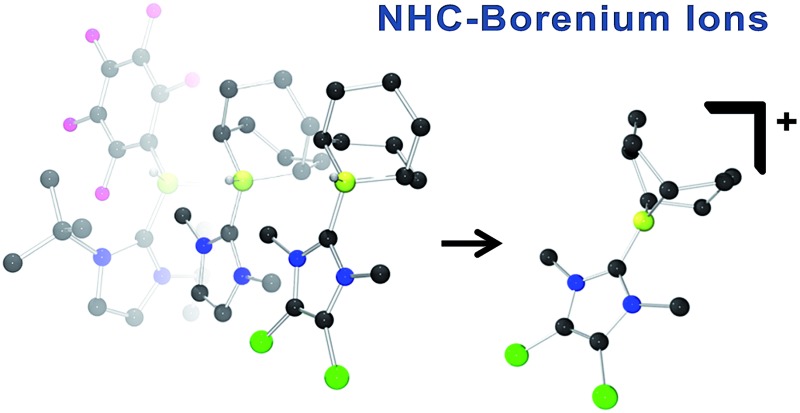
Room-temperature metal-free hydrogenation catalysis.

## Introduction

As the global consciousness awakens to the environmental and fiscal costs associated with energy and material-intensive chemical processes, the development of new and effective catalytic strategies grows ever more significant. The hydrogenation of unsaturated bonds is one such chemical transformation that is employed on a terrific industrial scale.^1^–^4^ Currently these processes employ highly effective transition metal catalysts despite oft associated high cost, toxicity and significant carbon footprint. These drawbacks have led to the intense pursuit of alternative or complimentary technologies. For example, hydrogenation catalysis by cheap and non-toxic transition metals such as iron^5^–^8^ and cobalt,^9^,^10^ as well as early metals such as titanium^11^,^12^ and calcium^13^ has drawn considerable attention. Our group^14^–^23^ and others^24^–^40^ have focused on main-group alternatives motivated by our report of metal-free hydrogen activation by a linked phosphino-borane.^41^ Indeed, soon after this initial report, we described the use of “frustrated Lewis pairs” (FLPs) in the catalytic hydrogenation of imines and protected nitriles.^23^ This prompted a flurry of developments in FLP hydrogenation catalysis. While substrate scope has since been dramatically broadened, the catalytic activities of FLP systems and the catalyst loadings required are not yet competitive with transition metal catalysts. Perhaps more importantly, the synthetic challenge of preparing electrophilic boranes limits the range of potential catalysts that are readily accessible for a systematic evaluation of structure–activity relationships. Indeed, although studies involving families of closely related FLP hydrogenation catalysts are rare,^29^,^38^,^39^,^42^ some examples in the literature suggests that subtle changes to FLP catalysts can have dramatic impact on activity and selectivity. For example, the group of Soós and co-workers has shown that substitution of one C_6_F_5_ in B(C_6_F_5_)_3_ with the bulkier mesityl group effects selectivity control through size exclusion.^34^,^35^ Moreover, careful choice of Lewis base in FLP hydrogenation catalysis has extended the substrate scope to include silyl enol ethers,^27^ olefins^19^,^33^ and most recently ketones and aldehydes.^43^,^44^

**Scheme 1:**
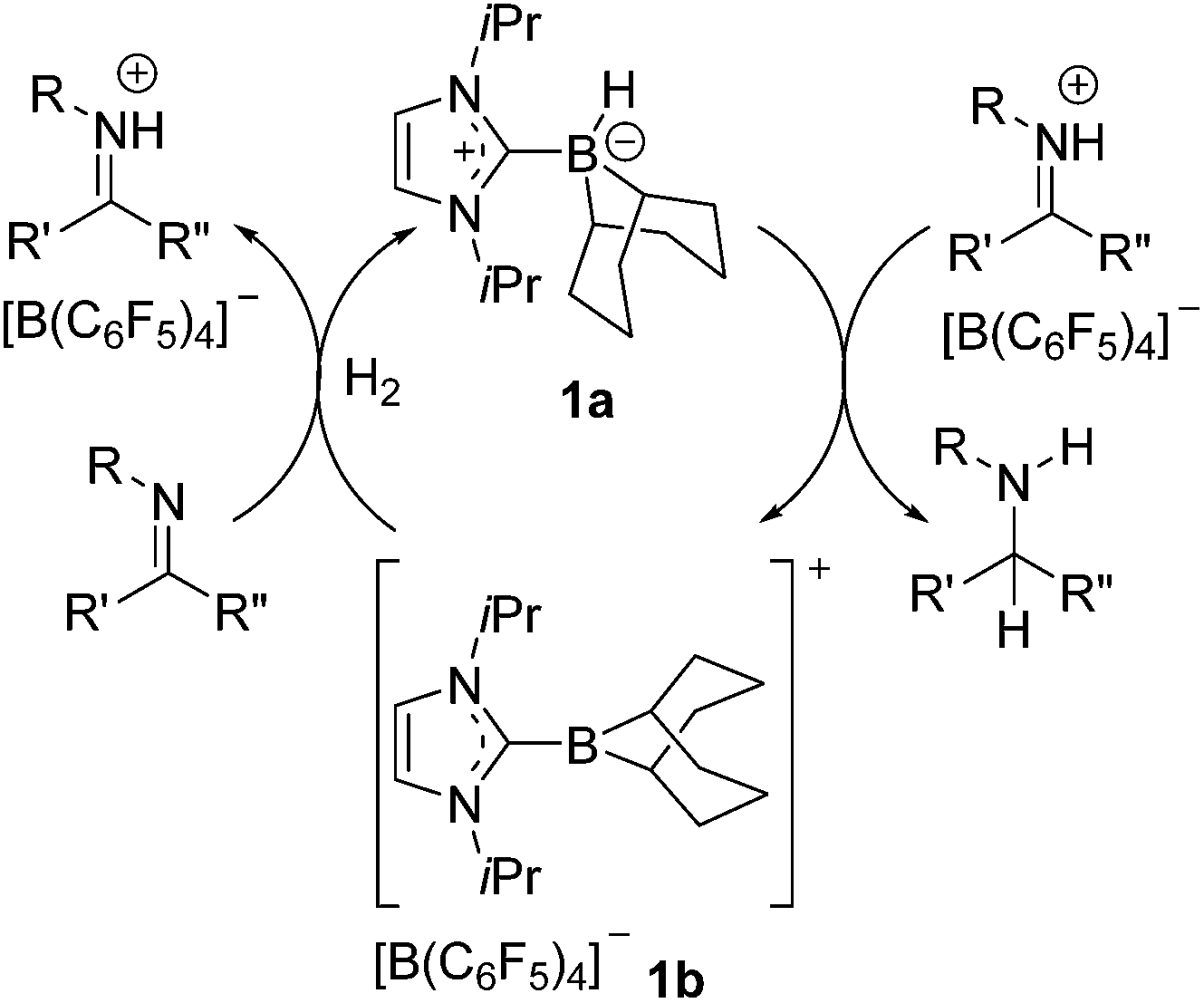
Hydrogenation catalysis by an NHC–borenium ion.

Borenium ions are three-coordinate boron cations.^45^–^47^ These relatively underexplored Lewis acids have attracted recent attention for use in catalysis^48^–^55^ and selective carboborations and haloborations.^56^–^66^ In an earlier communication, our group showed that an N-heterocyclic carbene-stabilized borenium salt [(IiPr)BC_8_H_14_] [B(C_6_F_5_)_4_] (IiPr = 1,3-di-iso-propylimidazol-2-ylidene) can be used as a catalyst for the metal-free hydrogenation of imines and enamines.^67^ In this case, the borenium cation and the imine act as an FLP to cleave H_2_. This affords an NHC–borane that delivers hydride to a transient iminium ion (Scheme 1). Borenium-based catalyst **1b** derives its Lewis acidity from a cationic charge rather than electron-withdrawing fluoroaryl groups on boron. Moreover, the precursor NHC–borane adduct is robust and easily accessible. During the review process of this paper Crudden and co-workers described triazolium derived borenium cations as catalysts.^80^

Herein, we exploit our previous findings to access a family of FLP catalysts. The reactivity of these borenium cations is evaluated in the metal-free hydrogenation catalysis of imines and N-heterocycles. This systematic study of the steric and electronic attributes of NHC–borenium catalysts provides insight into the structure–activity relationship of this new class of FLP hydrogenation catalyst.

## Results and discussion

**Scheme 2:**
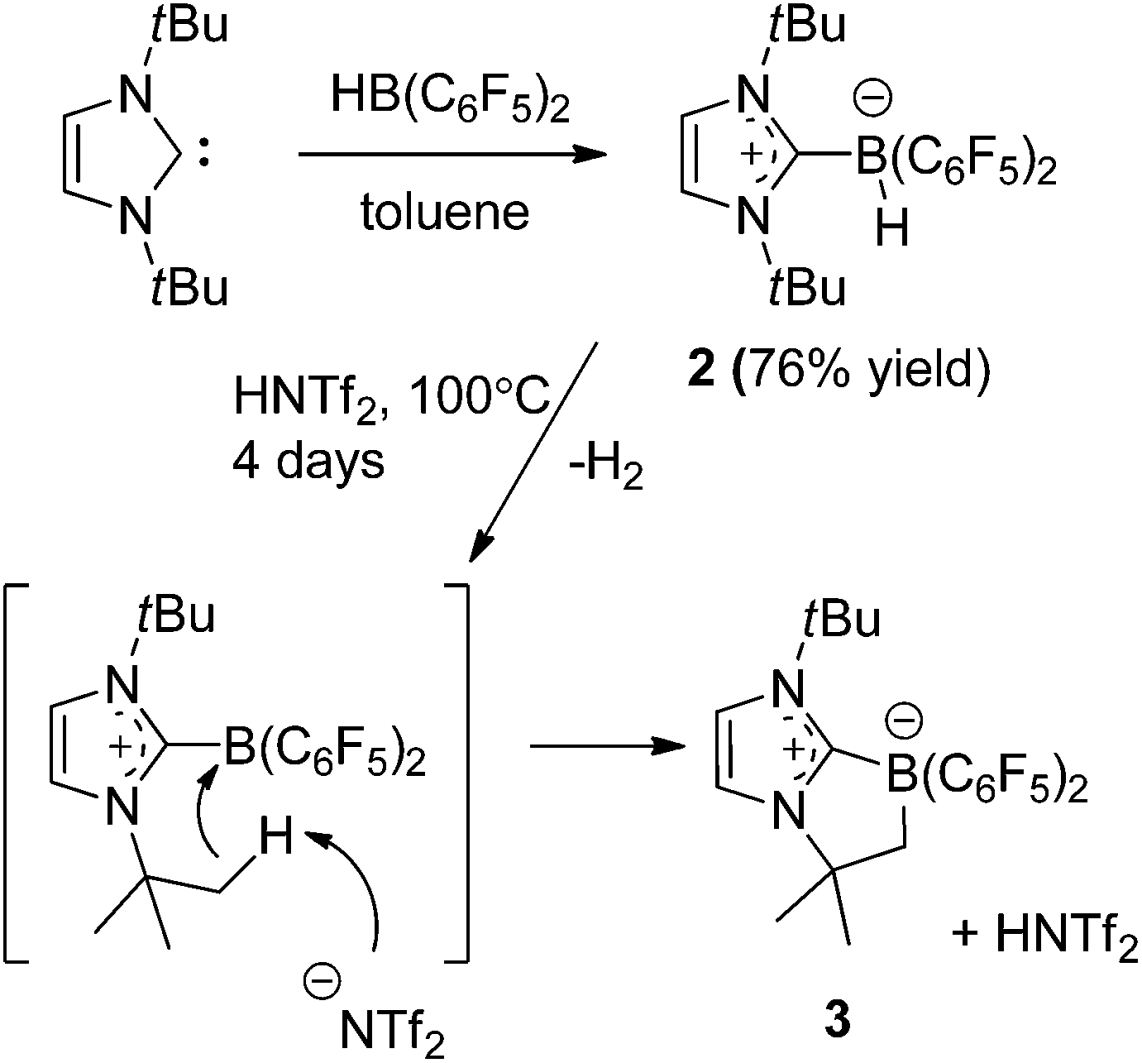
Synthesis and reactivity of NHC–borane **2**.

**Fig. 1:**
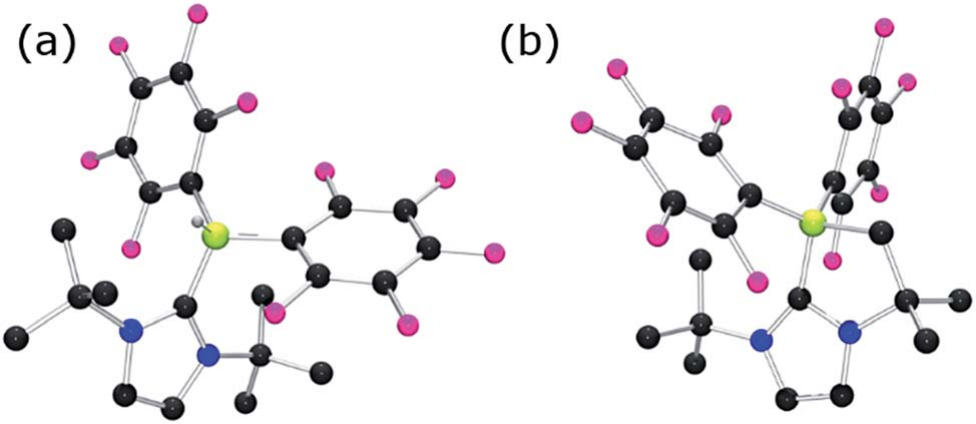
POV-ray depiction of (a) **2** and (b) **3**. C: black, B: yellow-green, N: blue, F: pink, H: grey. H-atoms except for borohydride omitted for clarity.

Our initial efforts to enhance reactivity with respect to previously reported catalyst **1b** focused on the incorporation of electron withdrawing C_6_F_5_ substituents in NHC–borenium ions. To this end, the reaction of 1,3-di-*tert*-butylimidazol-2-ylidene (I*t*Bu) with HB(C_6_F_5_)_2_ led to the formation of NHC–borane adduct (I*t*Bu)HB(C_6_F_5_)_2_**2** (Scheme 2), which was isolated in 76% yield. Crystallographic characterization revealed the anticipated pseudo-tetrahedral geometry about boron, an average C_NHC_–B bond length of 1.645(4) Å and an average B–C_C_6_F_5__ bond length of 1.639(4) Å (Fig. 1(a)). The expected upfield doublet for **2** is observed by ^11^B NMR spectroscopy at –22.9 ppm with a ^1^*J*_B–H_ coupling of 88 Hz. The ^1^H NMR and ^19^F NMR spectra indicate hindered rotation about the C_NHC_–B bond in **2** on the NMR time scale. Broad and inequivalent resonances were observed for the *tert*-butyl protons and fluorine atoms at room temperature; however, these were resolved upon cooling to –40 °C.

Attempts to generate an NHC–borenium ion derived from **2***via* treatment with the hydride abstraction reagents [Ph_3_C][B(C_6_F_5_)_4_], Me_3_SiOTf or HOTf showed no reaction. This stands in contrast to the facile hydride donation typically demonstrated by NHC–boranes.^68^,^69^ However, upon heating **2** with HNTf_2_ in toluene to >100 °C for four days the clean conversion to a new product was evident from the appearance of the ^11^B resonance at –14.8 ppm. ^1^H NMR spectroscopy showed sharp singlet resonances at 0.86 ppm and 1.04 ppm and a broad singlet resonance at 1.80 ppm integrating in a 9 : 6 : 2 ratio. These combined NMR data suggest the new species (CHN)_2_(*t*Bu) (CMe_2_CH_2_)CB(C_6_F_5_)_2_**3** is derived from C–H activation of a *tert*-butyl substituent (Scheme 2). A crystallographic study of **3** confirmed its bicyclic nature (Fig. 1(b)). This species is similar to compounds (CHN)_2_(*t*Bu)(CMe_2_CH_2_)CBBr_2_ 70 and (CHN)_2_(*t*Bu)(CMe_2_CH_2_) CB(*t*Bu)Cl^71^ recently reported by Braunschweig and co-workers. The formation of **3** is thought to proceed *via* transient generation of a cation and subsequent C–H activation (Scheme 2). Similar borylations of aliphatic groups by donor stabilized borenium ions have been reported by Prokofjevs and Vedejs.^72^

**Scheme 3:**
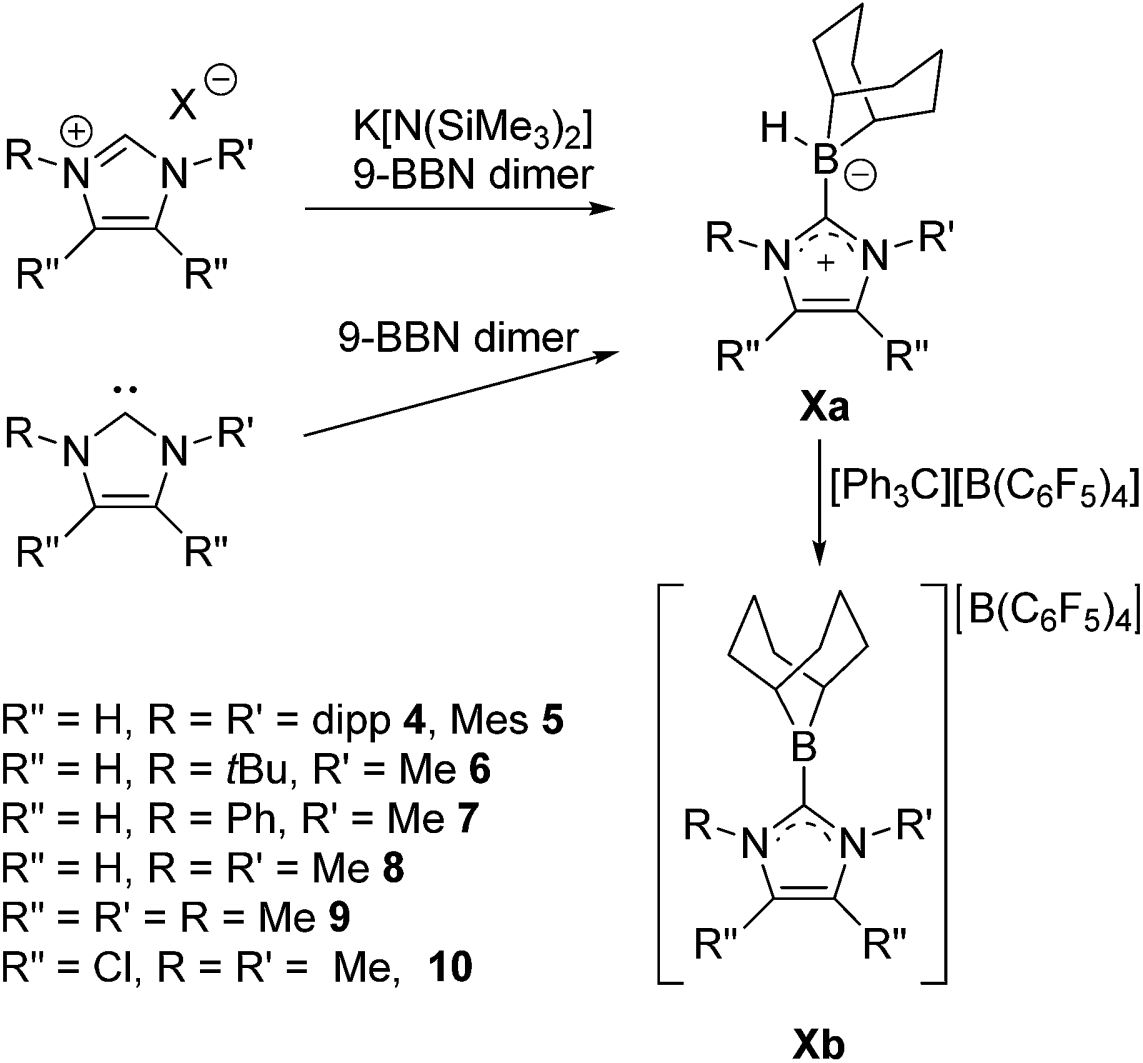
General syntheses of NHC–boranes **4a–10a** and generation of borenium salts **4b–10b**.

The C–H activation that yields **3** suggests that the proposed C_6_F_5_-substituted borenium ion derived from **2** is too Lewis acidic for application in catalysis. This prompted us to further examine 9-BBN based borenium cations. To this end 9-BBN was reacted with 1,3-bis(2,6-di-iso-propylphenyl)imidazol-2-ylidene (Idipp) at 60 °C for one hour to afford Idipp–borane adduct **4a** in 79% yield (Scheme 3). Compound **4a** exhibits a broad ^11^B NMR signal at –15.3 ppm. Reaction of **4a** with [Ph_3_C][B(C_6_F_5_)_4_] at 45 °C overnight results in the generation of Ph_3_CH and the quantitative conversion of the NHC–borane to a new species as evidenced by ^11^B NMR signals at 82.6 ppm and –16.6 ppm. These are consistent with the formation the borenium–borate salt [(Idipp)BC_8_H_14_][B(C_6_F_5_)_4_] **4b**. Alternatively, treatment of **4a** with *t*BuN=CHPh and the addition of a stoichiometric equivalent of [*t*Bu_3_PH]][B(C_6_F_5_)_4_] results in generation of **4b** with concurrent reduction of the imine as evidenced by ^1^H NMR spectroscopy. This observation prompted efforts to employ **4b** in an FLP hydrogenation of *t*BuN=CHPh. However combination of excess *t*BuN=CHPh and **4b** under 102 atm H_2(g)_ showed no evidence of reduction of the imine (Table 1, entry 2). Thus while the steric bulk of **4a** does not deter hydride delivery, it does preclude H_2_ activation by the corresponding borenium/imine FLP.

**Table 1:** Catalyst screening for the hydrogenation of *N*-benzylidene-*tert*-butylamine^*a*^

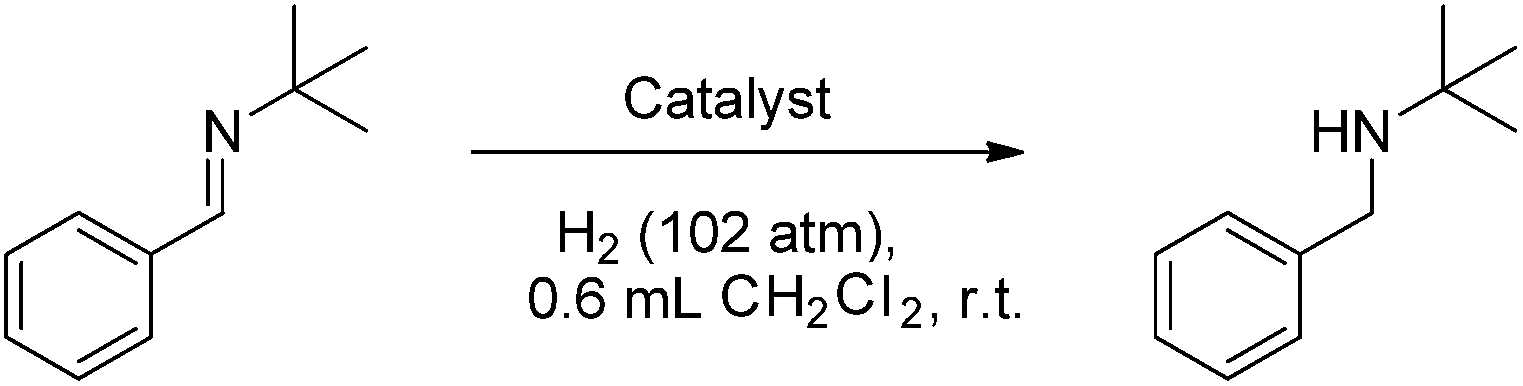		
Entry	Cat. (mol%)	Yield^*b*^ (%)
1	**1b** (1)	35
2	**4b** (5)	0
3	**5b** (1)	0
4	**6b** (1)	Trace
5	**7b** (1)	100
6	**7b** (0.5)	35
7	**8b** (1)	100
8	**8b** (0.5)	67
9	**9b** (0.5)	21
10	**10b** (0.5)	100
11	**10b** (0.25)	100
12	**10b** (0.1)	47
13	**10b** (0.15)	100 (83)^*c*^

^*a*^Borenium salts were generated *in situ* by addition of [Ph_3_C][B(C_6_F_5_)_4_] to the corresponding borohydride precursor. Isolated **10b** was used in entries 11–13.

^*b*^Determined by ^1^H NMR spectroscopy, isolated yields in parentheses. All reaction times were 30 min, except:

^*c*^2 h reaction time.

**Fig. 2:**
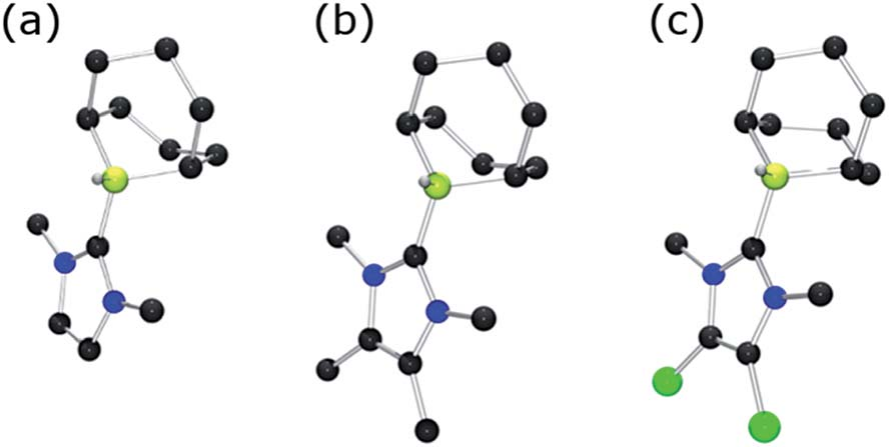
POV-ray depiction of (a) **8a** (b) **9a** (c) **10a**; C: black, B: yellow-green, N: blue, H: grey, Cl: green. H-atoms except BH omitted for clarity.

To further probe the steric and electronic factors impacting on the reactivity of NHC–borenium cations, a series of NHC-9-BBN adducts were prepared exercising judicious variation of the NHC. This was achieved by either directly reacting 9-BBN dimer with the isolated carbene or by reacting 9-BBN dimer with a carbene generated *in situ* through the combination of an imidazolium salt with K[N(SiMe_3_)_2_]. This latter one-pot approach is similar to that described by Brahmi *et al.* to prepare a series of NHC–BH_3_ compounds.^73^ A series of seven adducts including ((R′CNR)_2_C)HBC_8_H_14_ (R′ = H, R = dipp **4a**, Mes **5a**,^74^ Me **8a**;^50^ R = Me R′ = Me **9a**, Cl, **10a**) and ((HC)_2_(NMe)(NR)C)HBC_8_H_14_ (R = *t*Bu, **6a**, Ph **7a**) were prepared (Scheme 3). The NHC–borane adducts **4a–10a** were isolated and purified *via* recrystallization from pentane or toluene in yields ranging from 72–95%. The spectral data reported for these compounds were as expected and crystallographic data for **5a**, **7a**, (see ESI†) and **8a–10a** (Fig. 2) further corroborated these formulations.

**Fig. 3:**
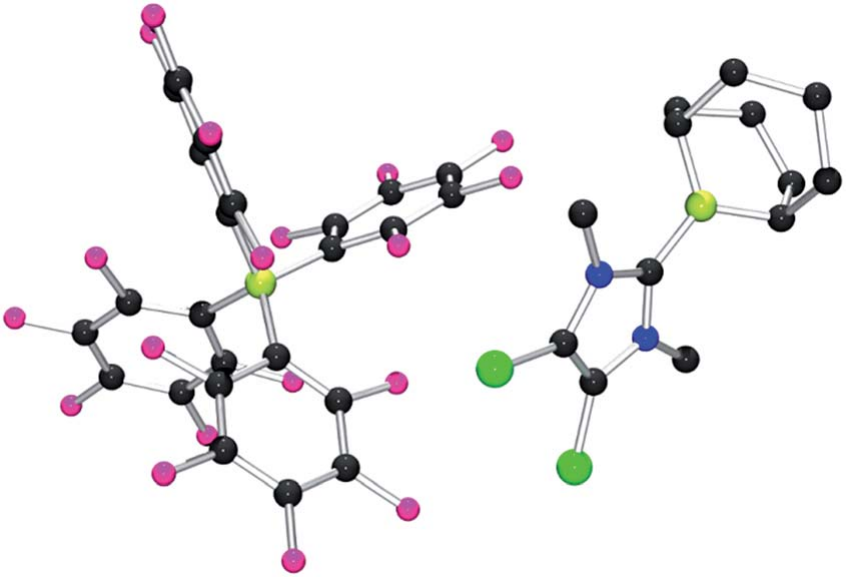
POV-ray depiction of **10b**. C: black, B: yellow-green, N: blue, F: pink, Cl: green. H-atoms omitted for clarity.

Each of these adducts reacts with [Ph_3_C][B(C_6_F_5_)_4_] to give the corresponding borenium salts [((R′CNR)_2_C)BC_8_H_14_] [B(C_6_F_5_)_4_] (R′ = H, R = dipp **4b**, Mes **5b**,^75^,^76^ Me **8b**; R = Me R′ = Me **9b**, Cl, **10b**) and [((HC)_2_(NMe)(NR)C)BC_8_H_14_] [B(C_6_F_5_)_4_] (R = *t*Bu, **6b**, Ph **7b**) concomitant with the generation of a stoichiometric amount of Ph_3_CH (Scheme 3). The most diagnostic spectroscopic change in each case is the appearance of a broad ^11^B resonance in the range of 81–88 ppm attributable to a three-coordinate B center. The expected resonances for the [B(C_6_F_5_)_4_]^–^ anion were seen at –16.7 ppm. The species **10b** was isolated as colorless crystals in 72% yield *via* recrystallization from CH_2_Cl_2_/pentane at –35 °C. Crystallographic data (Fig. 3) revealed trigonal planar geometry about the B center in the cation with a B–C_NHC_ bond length of 1.5768(3) Å similar to that observed for **1b** (1.580(3) Å).^67^

For comparative purposes the phosphine–borane adduct (Me_3_P)(HBC_8_H_14_) (**11**) was also synthesized and isolated as colorless crystals in 82% yield. The ^11^B NMR signal was observed at –14.9 ppm and exhibited both B–H coupling of 88 Hz and B–P coupling of 48 Hz. The ^31^P{^1^H} resonance for **11** is at –13.0 ppm and possesses similar B–P coupling. Single crystal X-ray diffraction confirmed the formulation (see ESI†). In contrast to the carbene complexes described above, treatment of **11** with stoichiometric [Ph_3_C][B(C_6_F_5_)_4_] gave a complex mixture of products as evidenced by ^31^P{^1^H} and ^11^B NMR-spectroscopy.

### Hydrogenation catalysis

Compounds **4b–10b** were tested for catalytic activity using the hydrogenation of *t*BuN=CHPh as a comparative screen. A solution of each was generated *in situ*, added to the imine substrate and pressurized with 102 atm H_2(g)_ for 30 minutes. After the reaction, the extent of conversion to amine was assessed by ^1^H NMR spectroscopy. These data reveal an inverse correlation between the steric demands of the NHC and the hydrogenation activity of the borenium catalyst (Table 1). The bulkiest catalyst **5b** shows no catalytic activity, while the slightly less bulky catalyst **6b** shows only trace conversion of imine to amine at 1 mol% catalyst loading after 30 minutes of reaction time. A sharp increase of catalytic activity is observed as the steric demands of the catalyst are further reduced. The previously reported catalyst **1b** allows for 35% conversion to amine at 1 mol% catalyst loading after 30 minutes while catalysts **7b** and **8b** show quantitative conversion.

Reducing the loadings of these catalysts to 0.5 mol% under otherwise identical conditions reduced the conversions and demonstrated that the least bulky catalyst **8b** effects 68% conversion while **7b** and **9b** reach only 35% and 21% conversion, respectively. In contrast, **10b** gave complete conversion. Even when the loading was dropped to 0.25 mol% under otherwise identical conditions **10b** gave complete conversion of imine to amine. Further reduction to 0.1 mol% gave 47% conversion representing a turn-over frequency (TOF) of 940 h^–1^. A slight increase of catalyst loading to 0.15 mol% and an extension of the reaction time to 2 h at room temperature under 102 atm H_2(g)_ led to complete conversion to *t*BuNHCH_2_Ph and the product could be isolated in 83% yield (Table 1, entry 13).

These observations reveal that the least sterically encumbered NHCs stabilize the most active borenium catalysts despite the fact that FLP reactivity hinges upon the steric protection of an acidic center. This suggests that the bulkier catalysts impede either H_2_ activation or hydride delivery in the catalytic cycle. Since bulky NHC–borane **4a** readily delivers hydride to an iminium ion it seems most likely that the bulkiest borenium ions are sterically prevented from generating the “encounter complex” with the imine that is required for H_2_ activation. Similarly diminished reactivity has been observed for FLPs incorporating excessively bulky boranes.^34^ It is noteworthy that computations suggest that a donor–boron distance of 4.2 Å is necessary to effect heterolytic cleavage of H_2_.^77^ Thus, it is reasonable to suggest that bulky peripheral substituents inhibit such a close approach.

Comparison of the isosteric catalysts **8b–10b** reveals that reduced donation from the NHC^78^ to the B center has a positive impact on the catalytic activity. This is thought to result from an increase in the Lewis acidity at B. That being said, further reduction of the donor ability of the stabilizing ligand^79^ jeopardizes the stability of the borenium cation as evidenced by the efforts to abstract hydride from **11**. Apparently the donor ability and steric demands of the NHC are suitably balanced in **10b** as it provides, to our knowledge, the highest TOF reported to date for the metal-free hydrogenation of imines. With the optimized catalyst **10b** in hand, a variety of N-containing unsaturated substrates were reduced affording products in high isolated yields (Table 2). In these cases a catalyst loading of 5 mol% was employed to ensure high conversions in 30 minutes and to overcome the impact of adventitious water. The imine *o*-ClC_6_H_4_CH=N*t*Bu, is readily reduced (Table 2, entry 1) as is *p*-(MeO_2_C)C_6_H_4_CH=N*t*Bu (Table 2, entry 2). The latter stands in contrast to previous FLP hydrogenations where sterically unencumbered esters preclude or inhibit reductions using the borane B(C_6_F_5_)_3_.^20^ While the steric demands of C_6_H_5_CH=NCHPh_2_ slow imine reduction (Table 2, entry 3), the aniline-derived ketimines Ph(Me)C=NPh and *p*-EtOC_6_H_4_(Me)C=NPh are readily hydrogenated to corresponding amines (Table 2, entries 4 and 5). In stark contrast, no hydrogenation of Ph(Me)C=NCH_2_Ph was observed (Table 2, entry 6). This was attributed to the greater basicity and lesser steric demands about the N-donor. 1,3,3-Trimethyl-2-methylideneindoline is hydrogenated to afford 1,2,3,3-tetramethylindoline (Table 2, entry 7), however 2,3,3-trimethylindolenine is not reduced (Table 2, entry 8). Nonetheless, in contrast to **1b**,^67^**10b** smoothly catalyzes the hydrogenation of 8-methylquinoline to 1,2,3,4-tetrahydro-8-methylquinoline (Table 2, entry 9), illustrating the subtlety of steric and electronic effects on substrate scope.

**Table 2:** Hydrogenation of N-containing substrates catalyzed by **10b**^*a*^

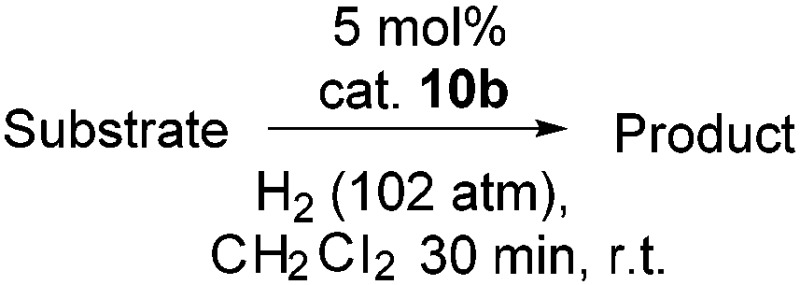			
Entry	Substrate	Product	Yield
**1**	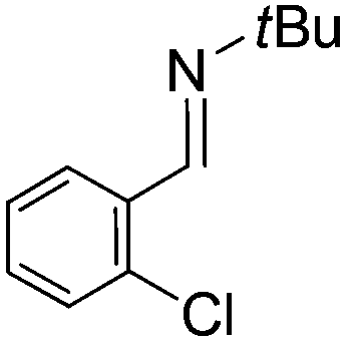	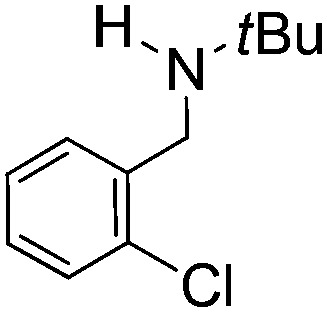	100^*b*^ (98)
**2**	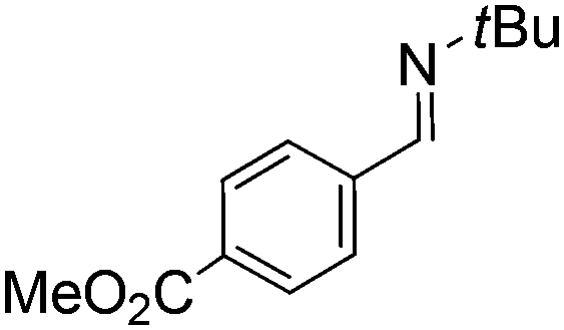	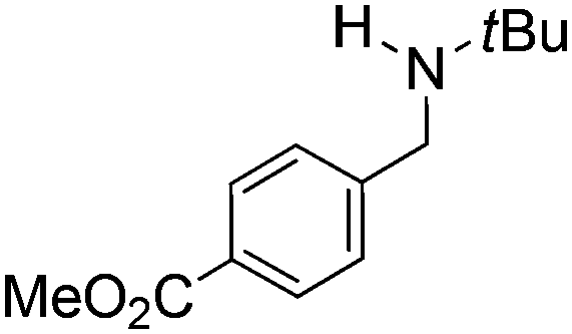	100 (82)
**3**	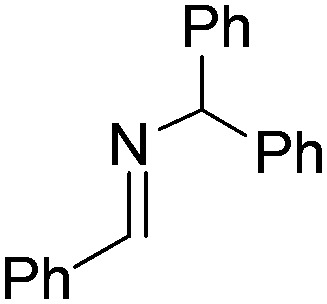	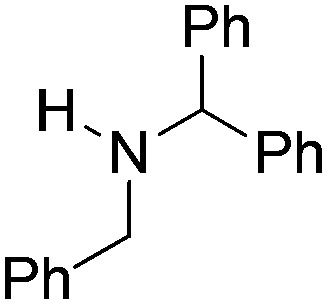	39
**4**	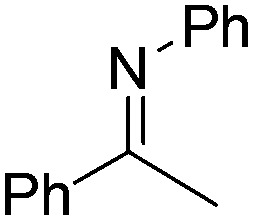	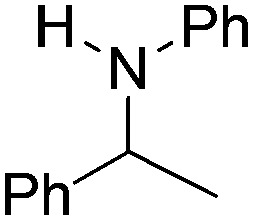	100 (71)
**5**	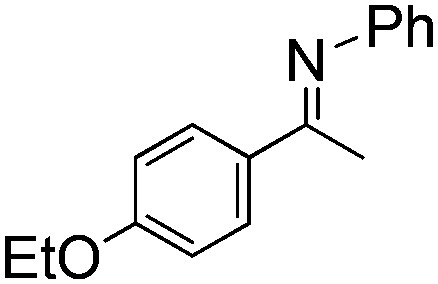	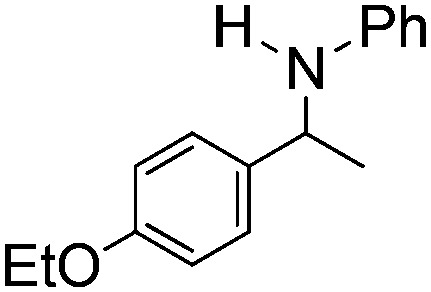	100 (95)
**6**	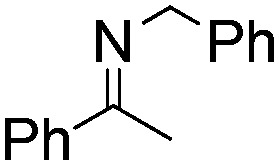	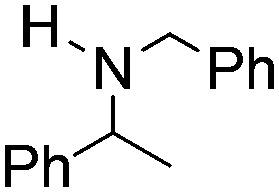	0
**7**	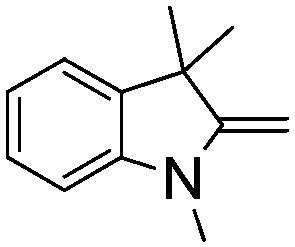	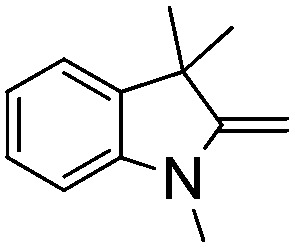	100 (91)
**8**	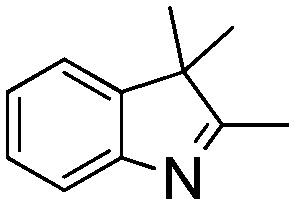	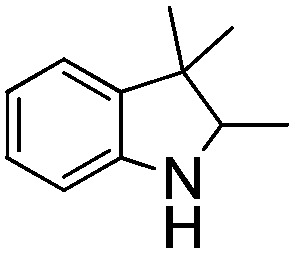	0
**9**	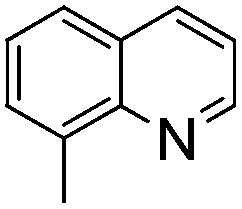	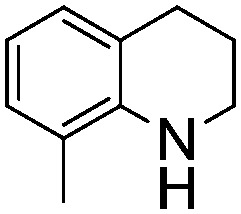	100 (87)

^*a*^Yields determined by ^1^H NMR spectroscopy, isolated yields in parentheses. All reactions were carried out using 0.500 mmol substrate in CH_2_Cl_2_. Reaction times were 30 minutes. Catalyst loadings: 5 mol% except:

^*b*^2.5 mol%.

## Conclusions

In this manuscript we have probed the electronic and steric parameters that impact on the ability of ligand stabilized borenium cations to act as metal-free hydrogenation catalysts. Although this catalysis proceeds *via* an FLP mechanism, perturbations that enhance the Lewis acidity at B or the steric demands of the NHC ligand can serve to deactivate the catalyst. At the same time, sterically unencumbered NHCs bearing electron withdrawing substituents enhance catalyst activity. Crudden and co-workers^80^ have very recently described related triazolium derived borenium cations and their use as catalysts for hydrogenation. Nonetheless, the present systematic examination of NHC stabilized borenium ion has led to catalysts that are highly efficient. Indeed the isolable catalyst **10b** is an effective catalyst for imine and N-heterocycle reduction at low catalyst loadings and it affords the highest TOF yet reported for metal-free hydrogenation catalysis. Efforts are continuing to systematically develop borenium-based metal-free hydrogenation catalysts and to further broaden their applications.

## Supplementary Material

Supplementary information

Crystal structure data
