# Supplementary material for: A family of N-heterocyclic carbene-stabilized borenium ions for metal-free imine hydrogenation catalysis
Source: Chem Sci. 2015 Jan 26;6(3):2010–5. doi: 10.1039/c4sc03675a (PMC5810240; doi:10.1039/c4sc03675a)
Supplement: Supplementary file 1 [file SC-006-C4SC03675A-s001.pdf]

Electronic Supplementary Material (ESI) for Chemical Science.
This journal is © The Royal Society of Chemistry 2015

N-heterocyclic Carbene-Stabilized Borenium Ions for Metal-Free Imine Hydrogenation Catalysis

Jeffrey M. Farrell, Roy T. Posaratnanathan and Douglas W. Stephan*

Department of Chemistry, University of Toronto, Toronto, ON, Canada.

Supplementary Information

Table of Contents

S2. General Considerations.

S4. Synthesis of compounds 2 and 3.

S5. Synthesis of NHC-boranes 4a to 10a.

S9. *In situ* generation of NHC-borenium ions 4b to 10b.

S25. Synthesis of 10b and 11.

S26. Synthesis of N-(*p*-methoxycarbonyl)benzyl-N-*tert*-butylamine.

S27. Procedures for Elevated Pressure Reductions.

S28. Hydrogenation Product Characterization Data.

S39. X-ray Crystallography.

S40. References.

General Considerations

All synthetic manipulations were carried out under an atmosphere of dry, O_2-free N_2 employing an MBraun glove box and a Schlenk vacuum-line. Pentane, dichloromethane, diethyl ether and toluene were purified with a Grubbs-type column system manufactured by Innovative Technology and dispensed into thick-walled glass Schlenk bombs equipped with Young-type Teflon valve stopcocks. Chloroform-*d* and dichloromethane-d_2 were each dried over CaH_2 and vacuum-transferred into a Young bomb. Tetrahydrofuran was dried over Na/benzophenone, vacuum-transferred into a Young bomb, and stored over 4 Å molecular sieves. All solvents were thoroughly degassed after purification (three freeze-pump-thaw cycles). NMR spectra were recorded at 25 °C on a Bruker Avance 400 MHz spectrometer, an Agilent DD2 500 MHz spectrometer or an Agilent DD2 600 MHz spectrometer unless otherwise noted. KHMDS, trimethylphosphine, trimethylsilyl trifluoromethanesulfonate, trifluoromethanesulfonic acid, *tert*-butylamine, methyl 4-formylbenzoate and 9-BBN dimer were obtained from Sigma-Aldrich and used without further purification. N-benzylidene-1,1-diphenylmethylamine and 1,3,3-trimethyl-2-methylideneindoline were obtained from Sigma-Aldrich. 8-methylquinoline and 2,3,3-trimethylindolenine were obtained from TCI America. The compounds bis(pentafluorophenyl)borane,[1] 1,3-di-*tert*-butylimidazol-2-ylidene,[2] 1,3-bis-(2,6-di-*iso*-propylphenyl)imidazol-2-ylidene,[3] 1-*tert*-butyl-3-methylimidazolium iodide,[4] 1-methyl-3-phenylimidazolium iodide,[5] 1,3-dimethylimidazolium iodide,[6] 4,5-dichloro-1,3-dimethylimidazolium iodide,[7] 1,2,3,4-tetramethylimidazolylidene,[8] N-benzylidene-*tert*-butylamine,[9] N-*o*-chlorobenzylidene-*tert*-butylamine,[10] N-(*p*-bromobenzylidene)aniline,[11] N-(1-phenylethylidene)aniline,[12] N-(1-(*p*-ethoxyphenyl)ethylidene)aniline,[13] N-(1-

phenylethylidene)benzylamine,[14] 9-chloro-9-borabicyclo[3.3.1]nonane[15] and bis(pentafluorophenyl)zinc·toluene[16] were prepared using literature methods. N-(*p*-methoxycarbonyl)benzyl-N-*tert*-butylamine was prepared as described herein. N-benzylidene-*tert*-butylamine and N-*o*-chlorobenzylidene-*tert*-butylamine were distilled from tri-*iso*-butylaluminum and stored in an inert atmosphere glovebox prior to use. Other liquid substrates were dried over 4Å molecular sieves. Solid substrates were dried in toluene solution over 4Å molecular sieves and isolated *via* recrystallization. Bis(trifluoromethane)sulfonimide was obtained from Apollo Scientific and used without further purification. Trityl tetrakis(pentafluorophenyl)borate was obtained from Nova Chemicals and used without further purification. 1,3-dimesitylimidazolium chloride was purchased from Strem Chemicals and used without further purification. Hydrogen gas (Grade 5.0) was obtained from Linde and purified through a Matheson Model 450B gas purifier. Chemical shifts are given relative to $SiMe_4$ and referenced to the residual solvent signal (^1H, ^{13}C) or relative to an external standard (^{11}B: 15% $(Et_2O)BF_3$; ^{19}F: 15% $(Et_2O)BF_3$; ^{31}P: 85% H_3PO_4). In some instances, signal and/or coupling assignment was derived from two-dimensional NMR experiments. Chemical shifts are reported in ppm and coupling constants as scalar values in Hz. Combustion analyses were performed in house employing a Perkin-Elmer CHN Analyzer. In some cases, elemental analyses were reproducibly low on carbon content, presumably due to the formation of boron carbides during combustion. Mass spectrometry was carried out using a Waters GCT Premier mass spectrometer with an EI source or a Jeol JMS T100—LC AccuTOF mass spectrometer with a DART source.

Synthesis of (1,3-di-*tert*-butylimidazol-2-ylidene)bis(pentafluorophenyl)borane (I^tBu_2)($HB(C_6F_5)_2$) (2).

In an inert atmosphere glovebox, 1,3-di-*tert*-butylimidazol-2-ylidene (223.7 mg, 1.241 mmol) and $HB(C_6F_5)_2$ (429.3 mg, 1.241 mmol) were weighed into vials. 5 mL toluene was added to each vial and the carbene solution was transferred to the stirring borane mixture. The reaction was stirred for 16 hours and then concentrated *in vacuo.* The residue was extracted with 5 mL dichloromethane, filtered through celite and layered with pentane at -35˚C to give colourless crystals which could be washed with 3 x 2 mL of pentane and dried *in vacuo* to give **2** in 76.4% yield (499 mg). ^{1}H NMR (600 MHz, CD_2Cl_2, 233 K): δ 7.29 (d, 1H, $^3J_{HH}$ = 2.4 Hz), 7.21 (d, 1H, $^3J_{HH}$ = 2.4 Hz), 4.00 (m, 1H, br), 1.63 (s, 9H), 1.23 (s, 9H). ^{11}B NMR (128 MHz, CD_2Cl_2, 298 K): δ –22.9 ($^1J_{BH}$ = 88Hz). ^{13}C{^{1}H} NMR (150 MHz, CD_2Cl_2, 233 K, partial): δ 118.32, 118.28, 61.9, 61.0, 30.3, 30.0. ^{19}F NMR (564 MHz, CD_2Cl_2, 233K): δ –130.5 (d, 1F, $^3J_{FF}$ = 24.5 Hz), –131.2 (d, 1F, $^3J_{FF}$ = 23.5 Hz), –131.8 (dd, 1F, $^3J_{FF}$ = 25.1 Hz, $^4J_{FF}$ = 7.5 Hz), –135.1 (dm, 1F, $^3J_{FF}$ = 24.5 Hz), -161.0 (t, 1F, $^3J_{FF}$ = 21 Hz), -161.9 (t, 1F, $^3J_{FF}$ = 21 Hz), -164.2 (tm, 1F, $^3J_{FF}$ = 22.3 Hz), -165.9 (tm, 2F, $^3J_{FF}$ = 22.5 Hz), -166.3 (tm, 1F, $^3J_{FF}$ = 22.5 Hz). HRMS (DART-EI+): mass [M-H] calc'd for $^{12}C_{23}{}^1H_{20}{}^{11}B^{19}F_{10}{}^{14}N_2$: 525.15599, found: 525.15575. Anal. Calcd. for $C_{23}H_{21}BF_{10}N_2$: C 52.50%, H 4.02%, N 5.32%. Found: C 52.06%, H 3.69%, N 5.75%.

Synthesis of compound 3.

In an inert atmosphere glovebox, (I^tBu_2)($BH(C_6F_5)_2$) (19.4 mg, 0.0369 mmol) was weighed into a vial. $HNTf_2$ (10.4 mg, 0.0369 mmol) in 0.6 mL toluene was added and the reaction mixture was transferred to an NMR tube. The tube was sealed and heated to 100˚C for four days. Upon cooling, colourless crystals formed. The supernatant was decanted and the crystals were washed

with toluene and pentane. The crystals were dried *in vacuo* to give **3** as a colourless solid (14.0 mg, 0.0267 mmol, 72.4% yield). ^{1}H NMR (400 MHz, tol-*d8*, 298 K): δ 6.29 (d, 1H, $^3J_{HH}$ = 2.1 Hz), 6.29 (d, 1H, $^3J_{HH}$ = 2.1 Hz), 1.80 (br, 2H) 1.04 (s, 6H), 0.86 (s, 9H). ^{11}B NMR (128 MHz, tol-*d8*, 298 K): –14.8. ^{13}C{^{1}H} NMR (101 MHz, tol-*d8*, 298 K, partial): δ 122.0, 113.7, 63.6, 58.7, 44.5 (br), 30.4, 29.4. ^{19}F NMR (376 MHz, tol-*d8*, 298K): δ –132.5 (br, 4F), –160.0 (t, 2F, $^3J_{FF}$ = 20 Hz), –164.7 (m, 4F). Anal. Calcd. for $C_{23}H_{19}BF_{10}N_2$: C 52.70%, H 3.65%, N 5.34%. Found: C 51.31%, H 3.90%, N 5.43%.

Synthesis of 1,3-bis(2,6-di-*iso*-propyl)imidazol-2-ylidene-9-borabicyclo[3.3.1]nonane (Idipp)(HBC_8H_{14}) (4a).

In an inert atmosphere glovebox, 1,3-bis(2,6-di-*iso*-propylphenyl)imidazol-2-ylidene (2 equivalents, 218.0 mg, 0.5610 mmol) was weighed into a vial and 9-BBN dimer (1 equivalent, 69.1 mg, 0.283 mmol) was weighed into a 50 mL Schlenk flask. 9-BBN was stirred in 10 mL toluene as 1,3-bis(2,6-di-*iso*-propylphenyl)imidazol-2-ylidene was transferred to the Schlenk with 10 mL toluene. The Schlenk flask was removed from the glovebox and heated to 60ºC for one hour while stirring. The solution was concentrated *in vacuo*. The residue was dissolved in 5 mL toluene, filtered through a Celite plug and recrystallized in a -35ºC glovebox freezer. The colourless crystals were washed with 3 x 1 mL cold pentane and dried *in vacuo* to give 225.1 mg (Idipp)(HBC_8H_{14}) (**4a**) (78.6% yield). ^{1}H NMR (400 MHz, C_6D_5Br, 298 K): δ 7.26 (t, 2H, $^3J_{HH}$ = 7.7 Hz), 7.10 (d, br, 4H, $^3J_{HH}$ = 7.7 Hz), 6.73 (s, 2H), 2.87 (m(7), 4H, $^3J_{HH}$ = 6.8Hz), 2.02-1.76 (m, br, 4H), 1.70-1.30 (m, br, 8H), 1.34 (d, 12H, $^3J_{HH}$ = 6.8 Hz), 1.03 (d, 12H, $^3J_{HH}$ = 6.8 Hz), 0.57 (m, br, 2H), B-*H* peak not observed. ^{11}B NMR (128 MHz, C_6D_5Br, 298 K): δ -15.30 (br). ^{13}C{^{1}H} NMR (101 MHz, CD_2Cl_2, 298 K, partial): 145.1, 134.7, 129.6, 123.1, 122.3, 36.2, 32.3,

28.3, 25.5, 25.5, 25.0, 21.7, 20.7 (br). HRMS (DART-EI+): mass [M-H] calc'd for $^{12}C_{35}{}^{1}H_{50}{}^{11}B^{14}N_2$: 509.40670, found: 509.40620. Anal. Calcd. for $C_{35}H_{51}BN_2$: C 82.33%, H 10.07%, N 5.49%. Found: C 82.04%, H 10.19%, N 5.40%.

Synthesis of NHC-9-BBN Adducts 5a, 6a, 7a, 8a and 10a.

In an inert atmosphere glovebox, imidazolium halide (1.000 mmol), 9-BBN dimer (0.1220g, 0.500 mmol), and KHMDS (0.2095g, 1.050 mmol) were weighed into a Schlenk flask bearing a magnetic stir bar. The flask was sealed with a septum and cooled to -78ºC under nitrogen. 25 mL of cold THF (-78ºC) was added while stirring. The solution was warmed to room temperature and stirred for 24h. Volatiles were removed *in vacuo*. The residue was extracted with pentane (3x5 mL) (**5a, 6a, 8a and 10a**) or washed with pentane (3x5 mL) and extracted with (3x5 mL) toluene (**7a**). The combined extracts were filtered through celite, concentrated to 5 mL in vacuo and recrystallized at -35ºC to afford colourless crystals. The supernatant was decanted and the crystals were washed with (3x2 mL) with cold pentane (**5a, 6a, 8a** and **10a**) or cold toluene (**7a**) and dried *in vacuo* to afford pure NHC-9-BBN.

1,3-bis(2,4,6-trimethylphenyl)imidazol-2-ylidene-9-borabicyclo[3.3.1]nonane (5a):[17]

0.3633g, 85% yield. ^{1}H NMR (400 MHz, CD_2Cl_2, 298 K): δ 6.96 (s, 4H), 6.85 (s, 2H), 2.35 (s, 6H), 2.15 (s, 12H), 1.63 – 1.00 (m, 12H), 0.21 (br, 2H), B-*H* not observed. ^{11}B NMR (128 MHz, CD_2Cl_2, 298 K): δ –15.84 (d, $^{1}J_{BH}$ = 78 Hz). ^{13}C {^{1}H} NMR (101 MHz, CD_2Cl_2, 298 K, partial): δ 139.3, 135.9, 135.8, 129.0, 122.4, 37.4, 32.2, 26.0, 24.3, 21.3, 18.3. HRMS (DART-EI+): mass [M-H] calc'd for $^{12}C_{29}{}^{1}H_{38}{}^{11}B^{14}N_1$: 425.31280, found: 425.31479.

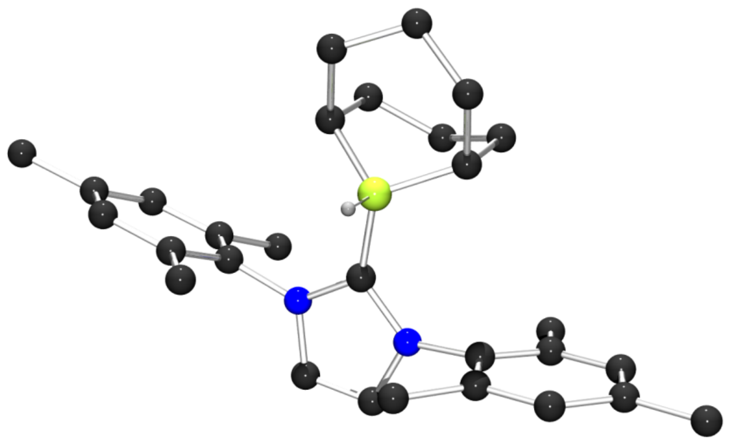

POV-ray depiction of 5a. C: black, B: yellow-green, N: blue, H: grey. Some H-atoms omitted for clarity.

1-*tert*-butyl-3-methylimidazol-2-ylidene-9-borabicyclo[3.3.1]nonane (6a):

0.1961g, 75% yield. ^{1}H NMR (400 MHz, CD_2Cl_2, 298 K): δ 7.11 (d, 1H, $^3J_{HH}$ = 2.1 Hz), 6.70 (d, 1H, $^3J_{HH}$ = 2.1 Hz,) 3.90 (s, 3H), 1.72 (s, 9H), 1.90 – 1.45 (m, 11H), 1.39 – 1.29 (m, 1H), 1.20 (br, 2H), B-*H* not observed. ^{11}B NMR (128 MHz, CD_2Cl_2, 298 K): δ –15.93 (d, $^1J_{BH}$ = 84 Hz). ^{13}C{^{1}H} NMR (101 MHz, CD_2Cl_2, 298 K, partial): δ 120.8, 118.6, 61.0, 38.6, 38.3, 31.4, 31.0, 25.2, 24.2. HRMS (DART-EI+): mass [M-H] calc'd for $^{12}C_{16}{}^1H_{28}{}^{11}B^{14}N_2$: 259.23455, found: 259.23509. Anal. Calcd. for $C_{16}H_{29}BN_2$: C 73.85%, H 11.23%, N 10.77%. Found: C 73.66%, H 11.65%, N 10.61%.

1-methyl-3-phenylimidazol-2-ylidene-9-borabicyclo[3.3.1]nonane (7a):

0.2023 g, 72 % yield. ^{1}H NMR (400 MHz, CD_2Cl_2, 298 K): δ 7.40-7.31 (m, 5H), 6.84 (d, 1H, $^3J_{HH}$= 2 Hz), 6.80 (d, 1H, $^3J_{HH}$= 2 Hz), 3.80 (s, 3H), 1.75 – 0.93 (m, 12H), 0.79 (br, 2H), B-*H* not observed. ^{11}B NMR (128 MHz, CD_2Cl_2, 298 K): δ –16.69 (d, $^1J_{BH}$ = 82 Hz). ^{13}C {^{1}H} NMR (101

MHz, CD_2Cl_2, partial): δ 140.2, 129.3, 129.0, 127.3, 122.1, 121.9, 37.2, 37.0, 31.4, 25.4, 24.9. HRMS (DART-EI+): mass [M-H] calc'd for $^{12}C_{18}{}^{1}H_{24}{}^{11}B^{14}N_2$: 279.20325, found: 279.20377. Anal. Calcd. for $C_{18}H_{25}BN_2$: C 77.15%, H 8.99%, N 10.00%. Found: C 77.04%, H 8.91%, N 9.69%.

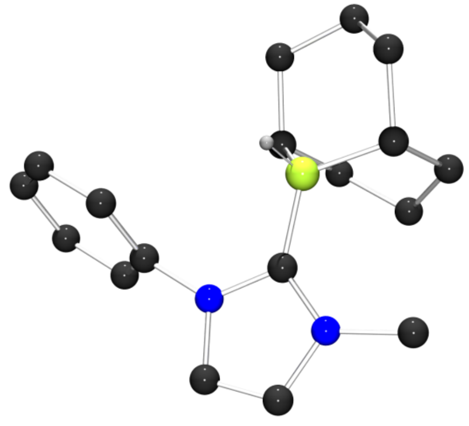

POV-ray depiction of 7a. C: black, B: yellow-green, N: blue, H: grey. Some H-atoms omitted for clarity.

1,3-dimethylimidazol-2-ylidene-9-borabicyclo[3.3.1]nonane (8a):[18]

0.2069 g, 95 % yield. ^{1}H NMR (400 MHz, CD_2Cl_2, 298 K): δ 6.71 (s, 2H), 3.61 (s, 6H), 1.90 – 1.40 (m, 12H) 0.45, (br, 2H), B-*H* not observed. ^{11}B NMR (128 MHz, CD_2Cl_2, 298 K): δ –16.88 (d, $^{1}J_{BH}$ = 81 Hz). ^{13}C {^{1}H} NMR (101 MHz, CD_2Cl_2, partial): δ 119.0, 37.1, 35.0, 31.8, 26.9, 26.4. HRMS (DART-EI+): mass [M-H] calc'd for $^{12}C_{13}{}^{1}H_{22}{}^{11}B^{14}N_2$: 217.18760, found: 217.18784. Anal. Calcd. for $C_{13}H_{23}BN_2$: C 71.58%, H 10.63%, N 12.84%. Found: C 71.63%, H 10.57%, N 12.94%.

4,5-dichloro-1,3-dimethylimidazol-2-ylidene-9-borabicyclo[3.3.1]nonane (10a):

0.2134g, 74 % yield. ^{1}H NMR (400 MHz, CD_2Cl_2, 298 K): δ 3.78 (s, 6H), 1.92 – 1.55 (m, 8H), 1.54 – 1.38 (m, 3H), 1.28 – 1.09 (m, 3H), B-*H* not observed. ^{11}B NMR (128 MHz, CD_2Cl_2, 298 K): δ –16.28 (d, $^{1}J_{BH}$ = 82 Hz). ^{13}C{^{1}H} NMR (101 MHz, CD_2Cl_2 partial): δ 116.6, 37.4, 34.2, 31.0, 25.1, 23.0. HRMS (DART-EI+): mass [M-H] calc'd for $^{12}C_{13}{}^{1}H_{20}{}^{11}B^{35}Cl_2{}^{14}N_2$: 285.10966, found: 285.11010. Anal. Calcd. for $C_{13}H_{21}BCl_2N_2$: C 54.40%, H 7.37%, N 9.76%. Found: C 54.41%, H 7.44%, N 9.84%.

Synthesis of 1,3,4,5-tetramethylimidazol-2-ylidene-9-borabicyclo[3.3.1]nonane ($(IMe_4)(HBC_8H_{14})$) (9a).

In an inert atmosphere glovebox, 1,3,4,5-tetramethylimidazol-2-ylidene (270 mg, 2.17 mmol) was dissolved in 5 mL toluene and added dropwise to a stirring solution of 9-borabicyclo[3.3.1]nonane dimer (244 mg, 1.00 mmol) in 5 mL toluene at room temperature. The reaction was stirred for 16 hours, concentrated *in vacuo* and extracted with 3 x 2mL pentane. The combined extracts were filtered through a celite plug and cooled to -35ºC to give colourless crystals. The supernatant was decanted and the crystals were washed with cold pentane (3 × 1 mL) and dried *in vacuo* to give $(IMe_4)(HBC_8H_{14})$ **(3-5)** (412 mg, 80.2% yield) as a white crystalline solid. ^{1}H NMR (400 MHz, CD_2Cl_2, 298 K): δ 3.63 (s, 6H), 2.09 (s, 6H), 1.91-1.75 (m, br, 3H), 1.74-1.52 (m, br, 5H), 1.49-1.36 (m, br, 3H), 1.27-1.13 (m, br, 3H), B-*H* not observed. ^{11}B NMR (CD_2Cl_2, 128 MHz): δ –16.84 (d, $^{1}J_{BH}$ = 81 Hz). ^{13}C{^{1}H} NMR (101 MHz, CD_2Cl_2, 298 K, partial): δ 123.8, 37.7, 32.9, 31.2, 25.1, 23.4, 22.5 (q, br, $^{1}J_{CB}$ = 41 Hz), 9.0. HRMS (DART-EI+): mass [M-H] calc'd for $^{12}C_{15}{}^{1}H_{26}{}^{11}B^{14}N_2$: 245.21890, found: 245.21962. Anal.

Calcd. for $C_{15}H_{27}BN_2$: C 73.18%, H 11.05%, N 11.38%. Found: C 72.97%, H 11.71%, N 11.28%.

***In situ* generation of NHC-borenium ions 4b to 10b.**

In an inert atmosphere glovebox, NHC-borane (0.025 mmol) and $[CPh_3][B(C_6F_5)_4]$ (0.0231g, 0.025 mmol) were weighed into vials. The reagents were combined in CD_2Cl_2 and ^1H, ^{11}B, ^{13}C and ^{19}F-NMR spectra were collected. **4b** and **5b** were reacted at 45ºC for 12 hours before spectra were collected.

[1,3-bis(2,6-di-*iso*-propyl)imidazol-2-ylidene-9-borabicyclo[3.3.1]nonane][tetrakis(pentafluorophenyl)borate] [(Idipp)(BC_8H_{14})] [$B(C_6F_5)_4$] (4b).

^1H NMR (500 MHz, CD_2Cl_2, 298 K, $HCPh_3$ omitted): δ 7.73 (s, 2H), 7.68 (t, 2H, $^3J_{HH}$ = 7.9 Hz), 7.45 (d, 4H, $^3J_{HH}$ = 7.9 Hz), 2.44 (m(7), 4H, $^3J_{HH}$ = 6.8 Hz), 1.95-1.72 (m, br, 6H), 1.33-1.08 (m, br, 8H), 1.30, (d, 12H, $^3J_{HH}$ = 6.8 Hz), 1.23 (d, 12H, $^3J_{HH}$ = 6.8 Hz). ^{11}B NMR (128 MHz, CD_2Cl_2, 298 K): δ 82.6 (br), –16.62 (s). $^{13}C\{^1H\}$ NMR (126 MHz, CD_2Cl_2, 298 K, partial, $HCPh_3$ omitted): δ 148.6 (dm, $^1J_{CF}$ = 244 Hz), 145.0, 138.7 (dm, $^1J_{CF}$ = 241 Hz), 136.7 (dm, $^1J_{CF}$ = 250 Hz), 133.3, 131.9, 129.3, 125.5, 34.9, 32.9 (br), 30.1, 26.0, 22.5, 22.3. ^{19}F NMR (CD_2Cl_2, 376 MHz): δ –133.0 (m, br, 8F, *o*–C_6F_5), –163.7 (t, 4F, *p*–C_6F_5, $^3J_{FF}$ = 20.5 Hz), –167.5 (t, br, 8F, *m*–C_6F_5, $^3J_{FF}$ = 18 Hz).

^{1}H NMR (500 MHz, CD_2Cl_2, 298 K):

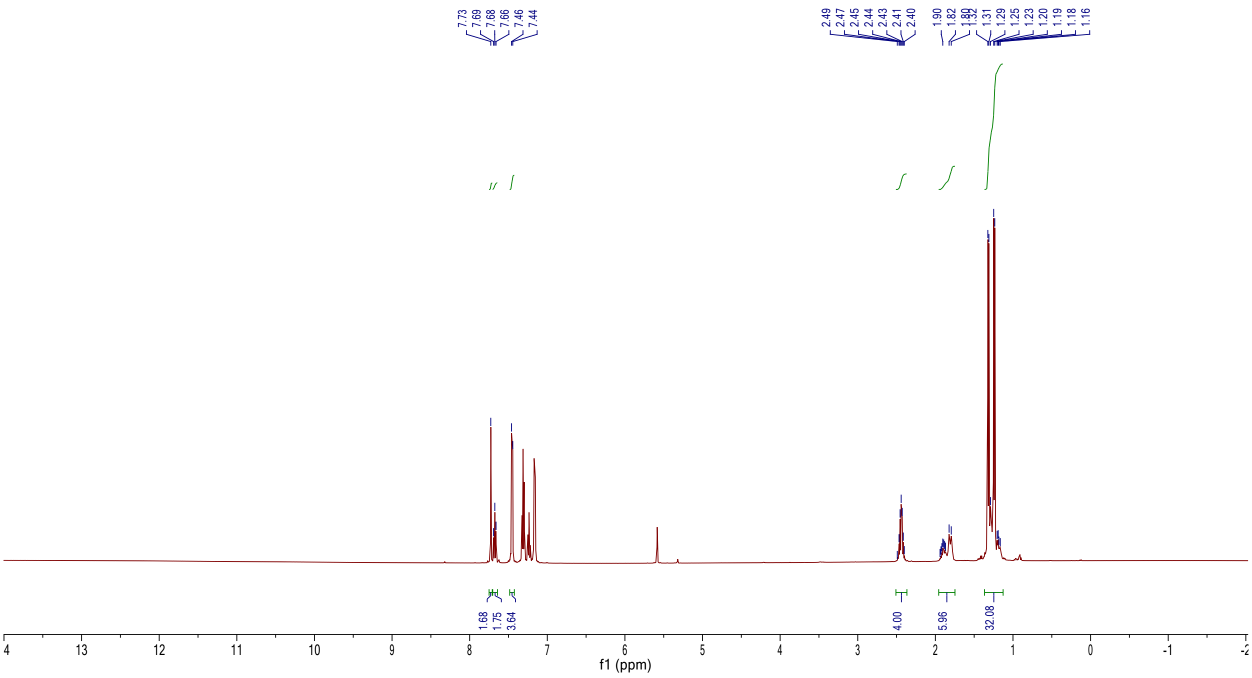

^{11}B NMR (128 MHz, CD_2Cl_2, 298 K):

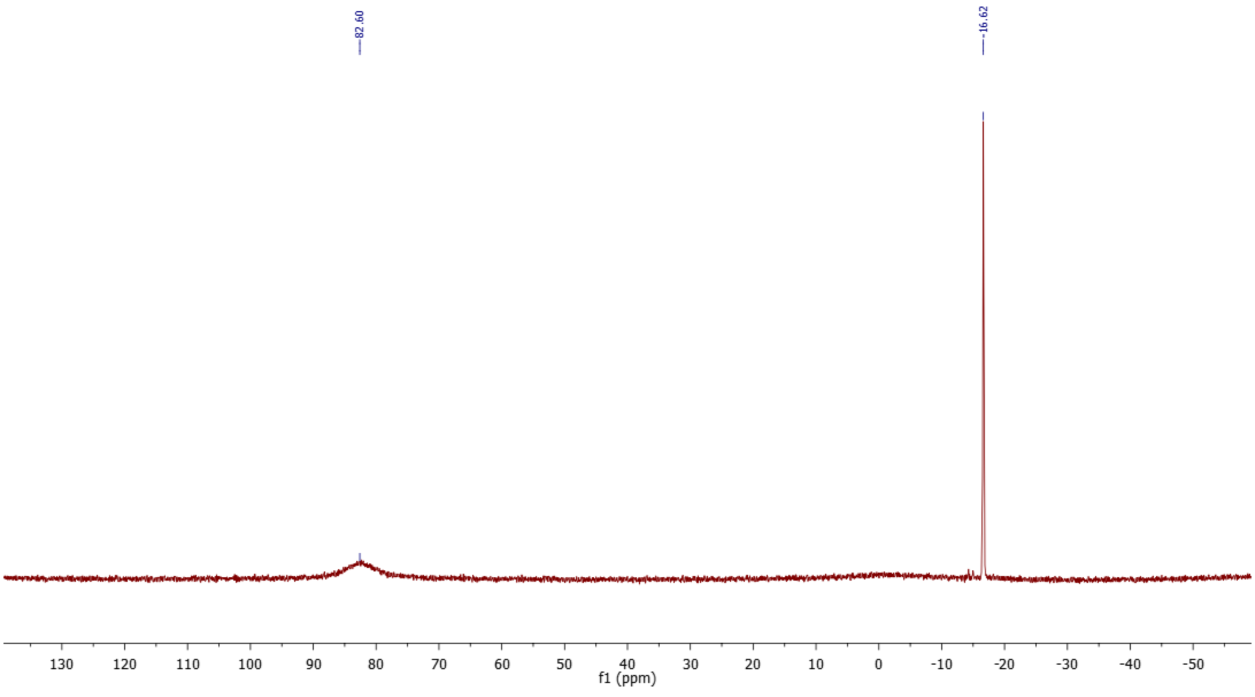

$^{13}C\{^1H\}$ NMR (126 MHz, CD_2Cl_2, 298 K):

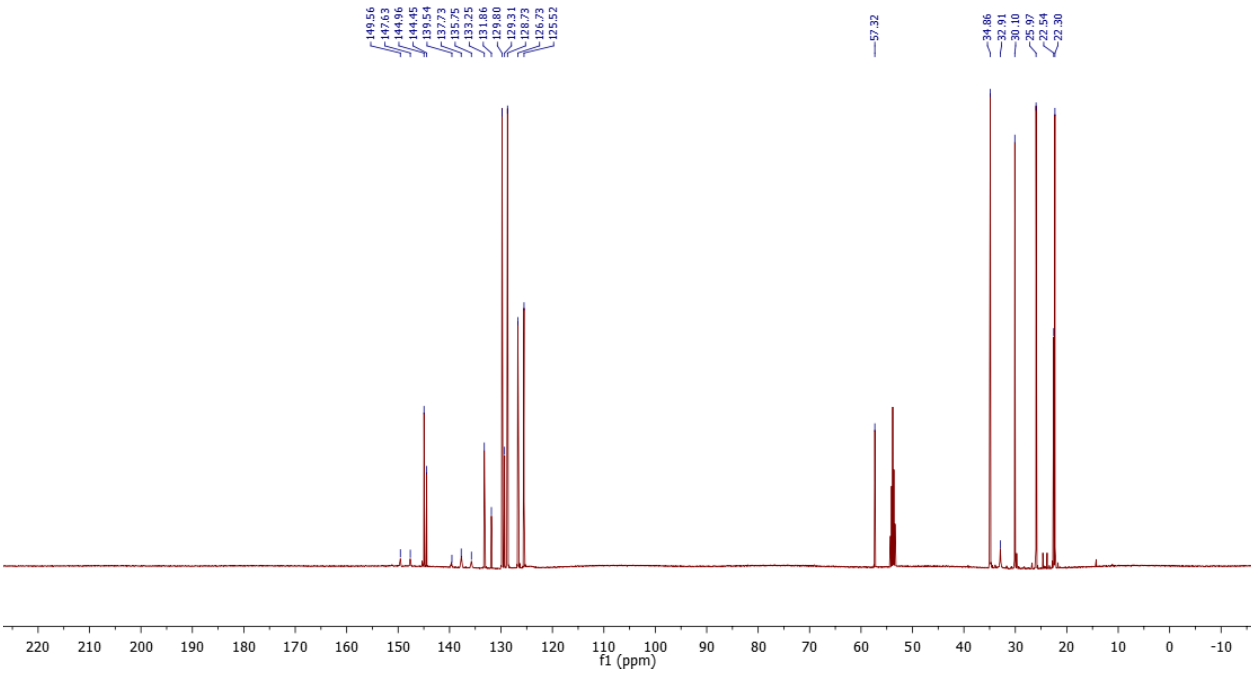

^{19}F NMR (CD_2Cl_2, 376 MHz, 298 K):

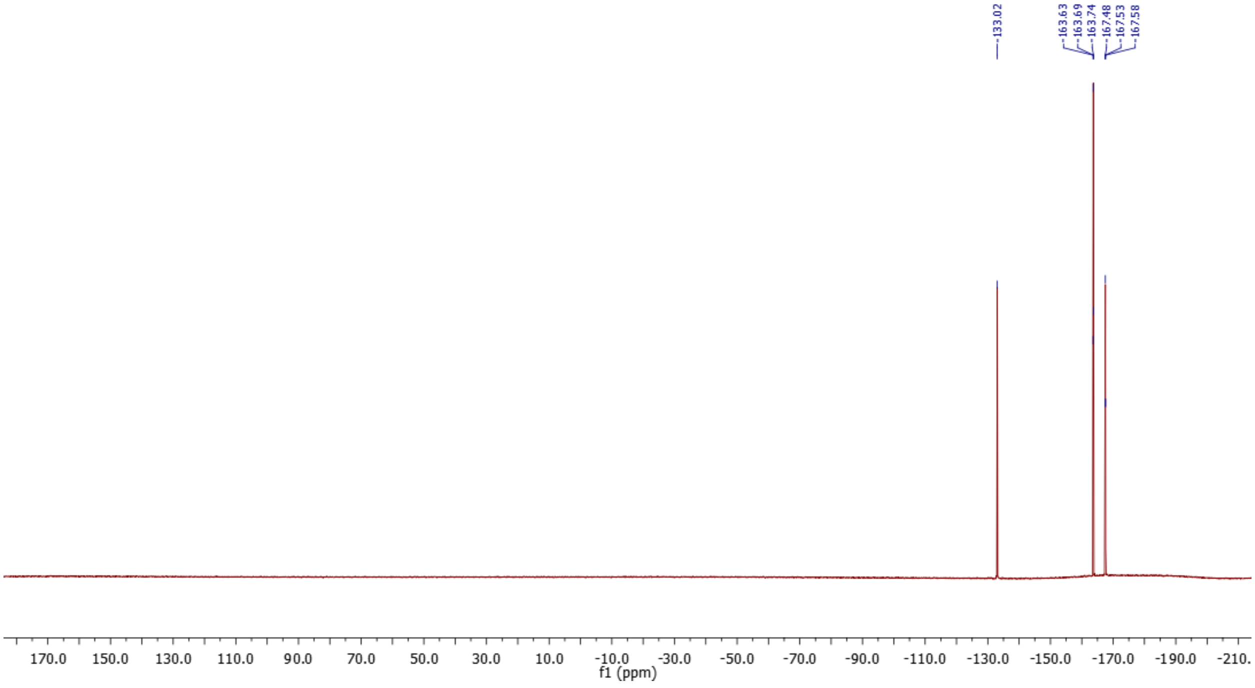

[1,3-dimesitylimidazol-2-ylidene-9-borabicyclo[3.3.1]nonane][tetrakis(pentafluorophenyl)borate] [(IMes)(BC_8H_{14})][$B(C_6F_5)_4$], (5b).

^{1}H NMR (500 MHz, CD_2Cl_2, 298 K, $HCPh_3$ omitted): δ 7.34 (s, 2H) 6.87 (s, 4H), 2.13 (s, 6H), 1.86 (s, 12H), 1.60-1.50 (m, br, 6H), 1.14 (br, 2H), 0.98-0.86 (m, br, 4H), 0.85-0.75 (m, br, 2H). ^{11}B NMR (CD_2Cl_2, 128 MHz, 298K): δ 84.40 (br), -16.66 (s). ^{13}C{^{1}H} NMR (126 MHz, CD_2Cl_2, 298 K, partial, $HCPh_3$ omitted): 148.6 (dm, $^1J_{CF}$ = 241 Hz), 143.1, 138.7 (dm, $^1J_{CF}$ = 244 Hz), 136.7 (dm, $^1J_{CF}$ = 244 Hz), 134.5, 131.8, 130.7, 127.8, 57.3, 35.2, 33.3 (br), 22.6, 21.3, 17.5. ^{19}F NMR (377 MHz, CD_2Cl_2, 298K): δ -133.1 (m, br, 8F, *o*–C_6F_5); -163.7 (t, 4F, *p*–C_6F_5, $^3J_{FF}$ = 20 Hz), -167.6 (t, br, 8F, *m*–C_6F_5, $^3J_{FF}$ = 17 Hz).

^{1}H NMR (500 MHz, CD_2Cl_2, 298 K):

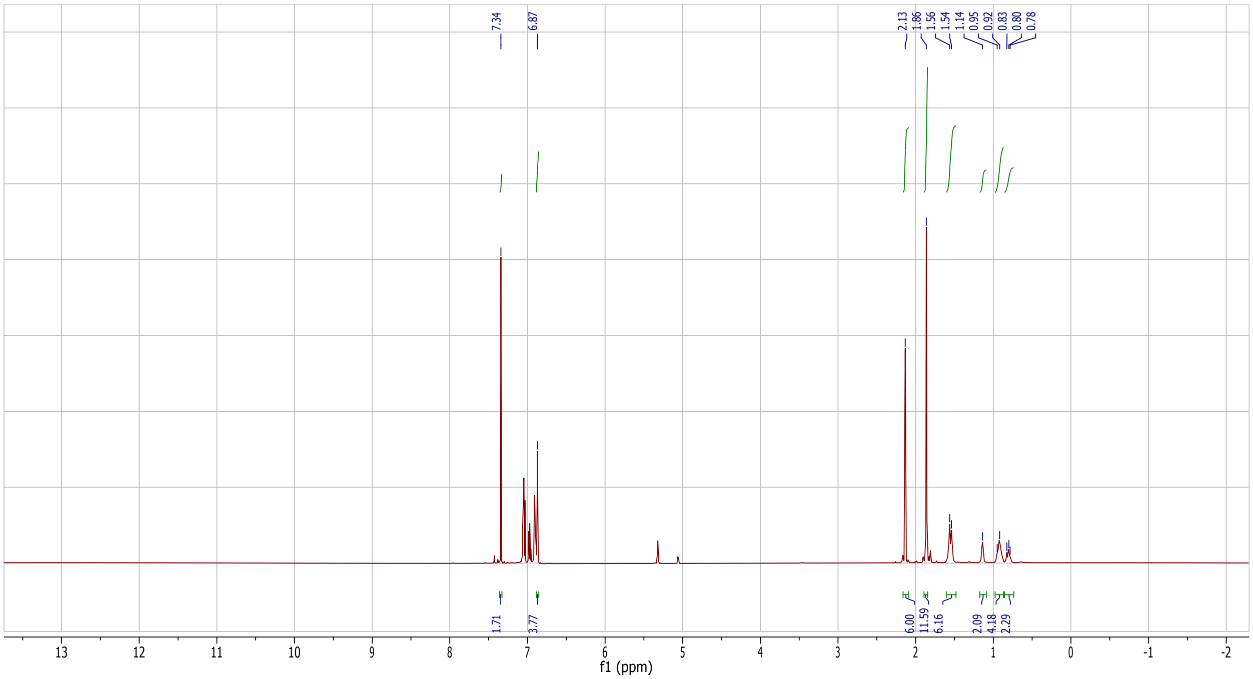

^{11}B NMR (CD_2Cl_2, 128 MHz, 298K):

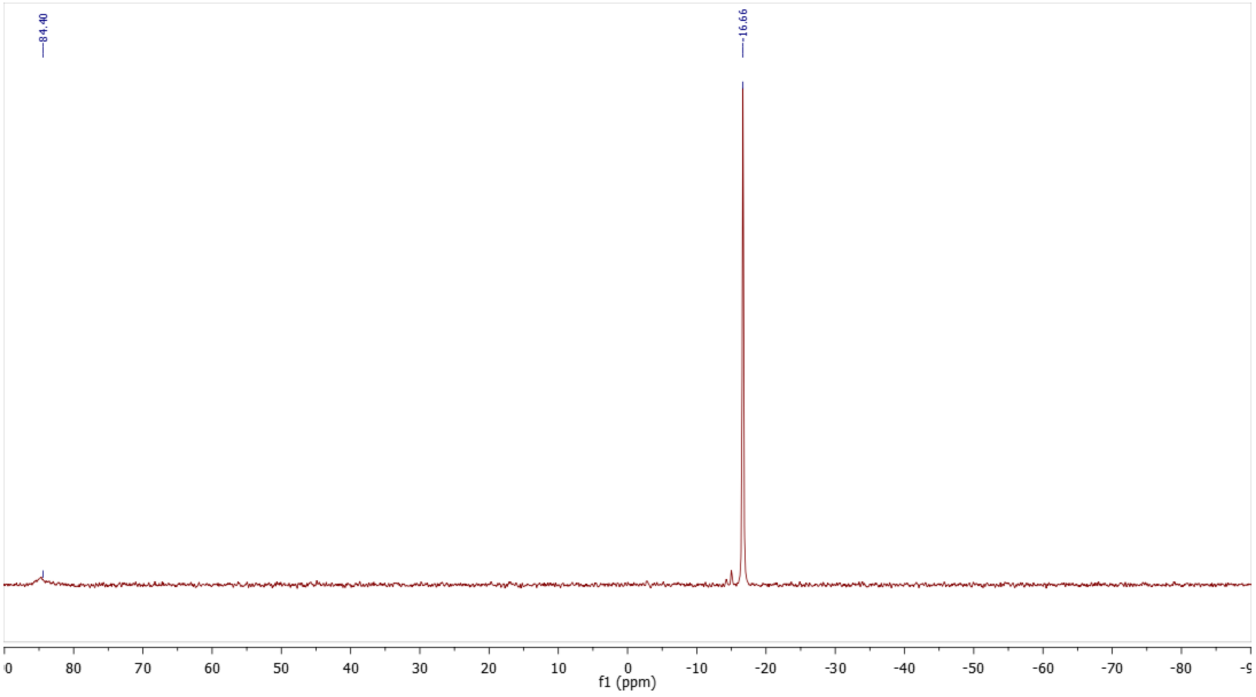

$^{13}C\{^1H\}$ NMR (126 MHz, CD_2Cl_2, 298 K):

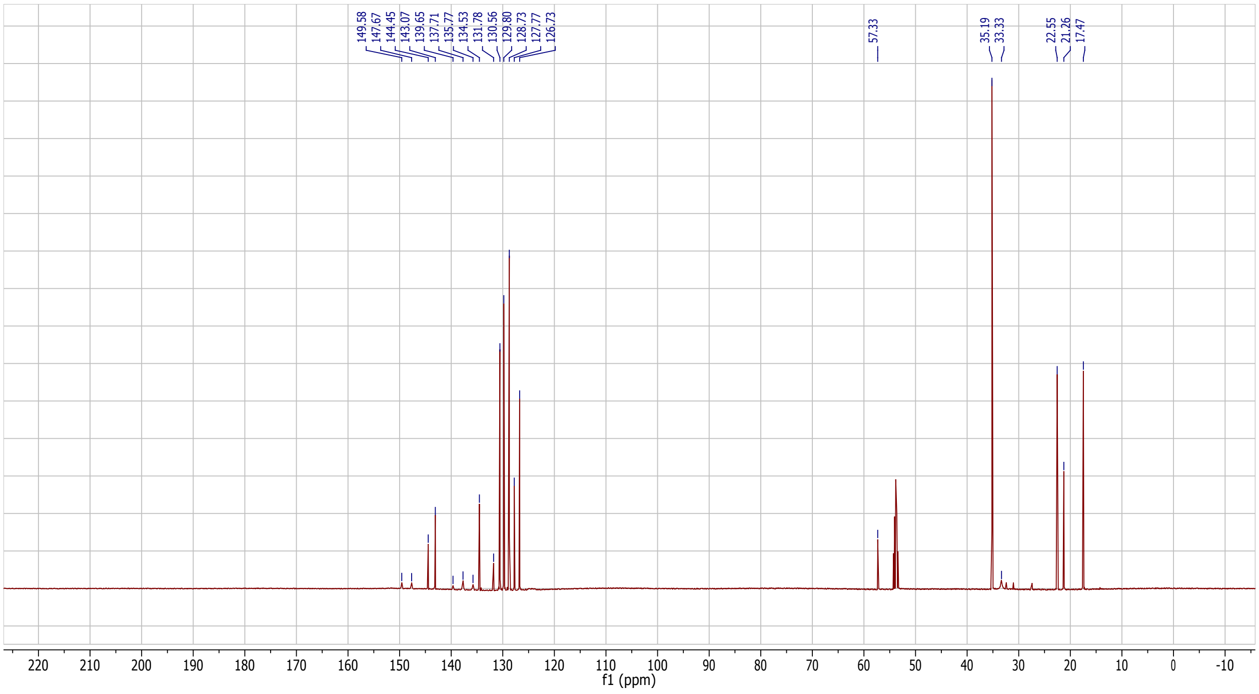

^{19}F NMR (377 MHz, CD_2Cl_2, 298K):

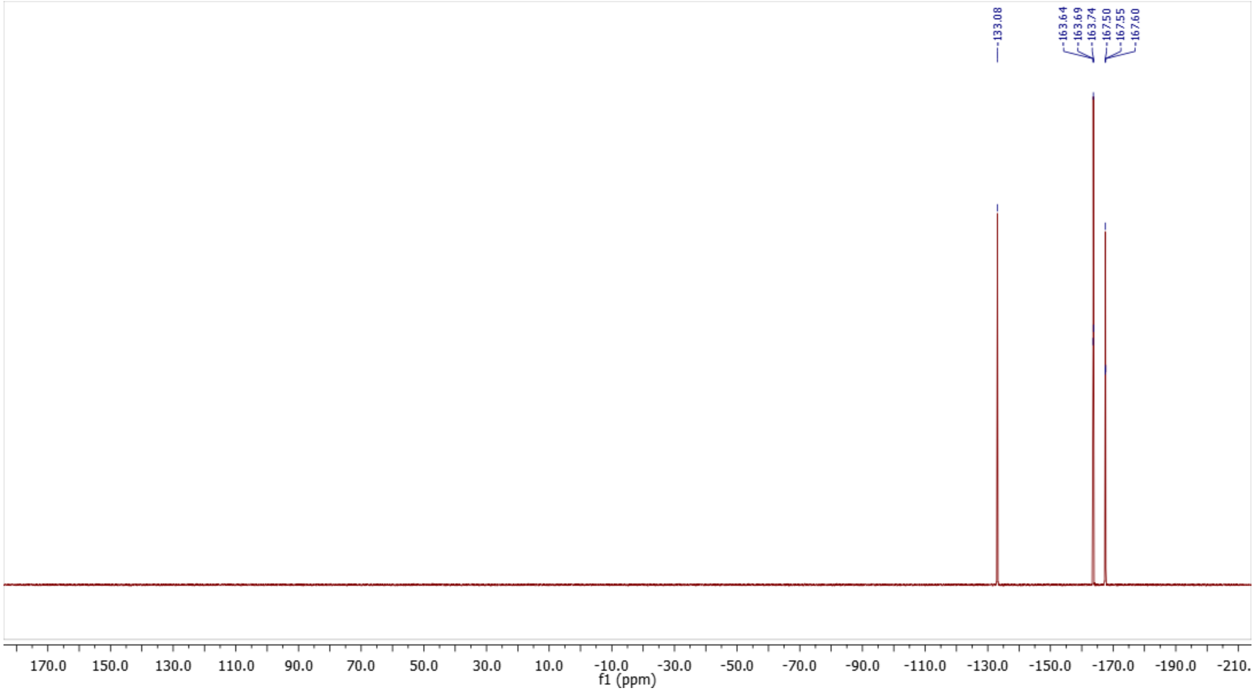

[1-*tert*-butyl-3-methylimidazol-2-ylidene-9-borabicyclo[3.3.1]nonane][tetrakis(pentafluorophenyl)borate] [(*t*-BuIMe)(BC_8H_{14})][$B(C_6F_5)_4$], (6b).

^{1}H NMR (400 MHz, CD_2Cl_2, 298K, $HCPh_3$ omitted): δ 7.47 (d, 1H, $^{3}J_{HH}$ = 2.0 Hz), 7.26 (d, 1H, $^{3}J_{HH}$ = 2.0 Hz), 3.91 (s, 3H, CH_3), 2.40-2.22 (m, 6H); 2.14-2.00 (m, 4H), 1.97 (br, 2H), 1.82-1.68 (m, 2H), 1.63 (s, 9H). ^{11}B NMR (128 MHz, CD_2Cl_2, 298K): δ 85.85 (br), -16.64 (s). ^{13}C NMR (101 MHz, CD_2Cl_2, 298K, partial, $HCPh_3$ omitted): δ 148.6 (dm, $^{1}J_{CF}$ = 241 Hz), 138.7 (dm, $^{1}J_{CF}$ = 242 Hz), 136.7 (dm, $^{1}J_{CF}$ = 244 Hz), 125.5, 121.9, 61.0, 57.3, 39.1, 36.6, 34.6, 31.9, 22.5. ^{19}F NMR (376 MHz, CD_2Cl_2, 298K): δ -133.1 (br, 8F, *o*–C_6F_5), -163.6 (t, 4F, $^{3}J_{FF}$ = 20 Hz, *p*–C_6F_5), -167.5 (t, 8F, $^{3}J_{FF}$ = 18 Hz, *m*–C_6F_5).

^{1}H NMR (400 MHz, CD_2Cl_2, 298K):

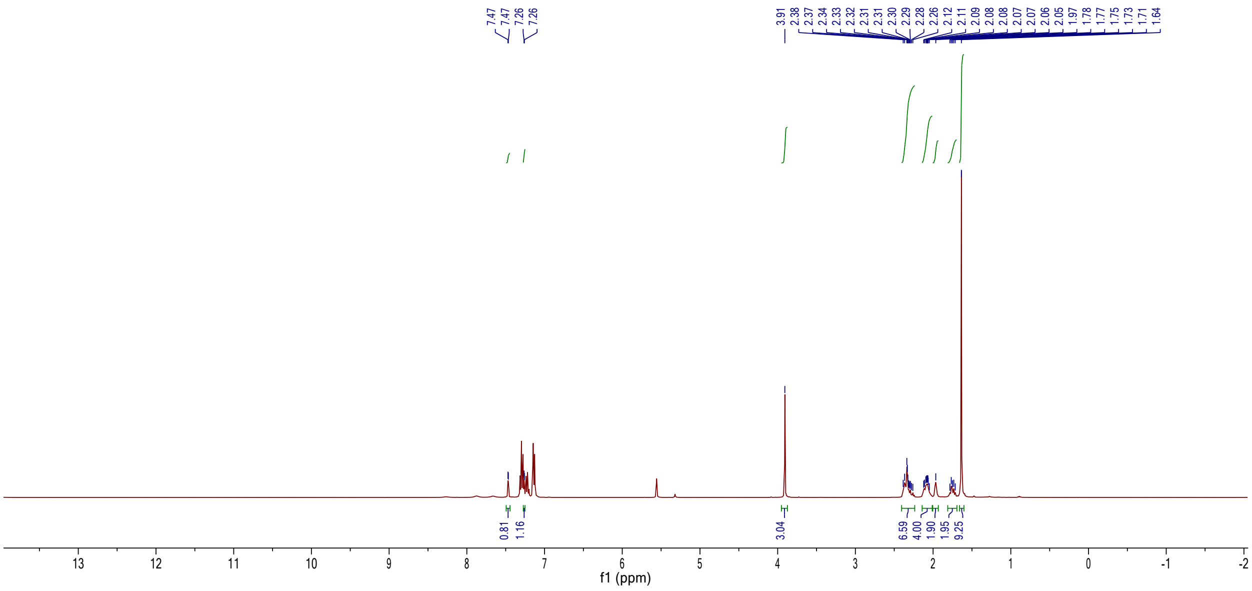

^{11}B NMR (128 MHz, CD_2Cl_2, 298K):

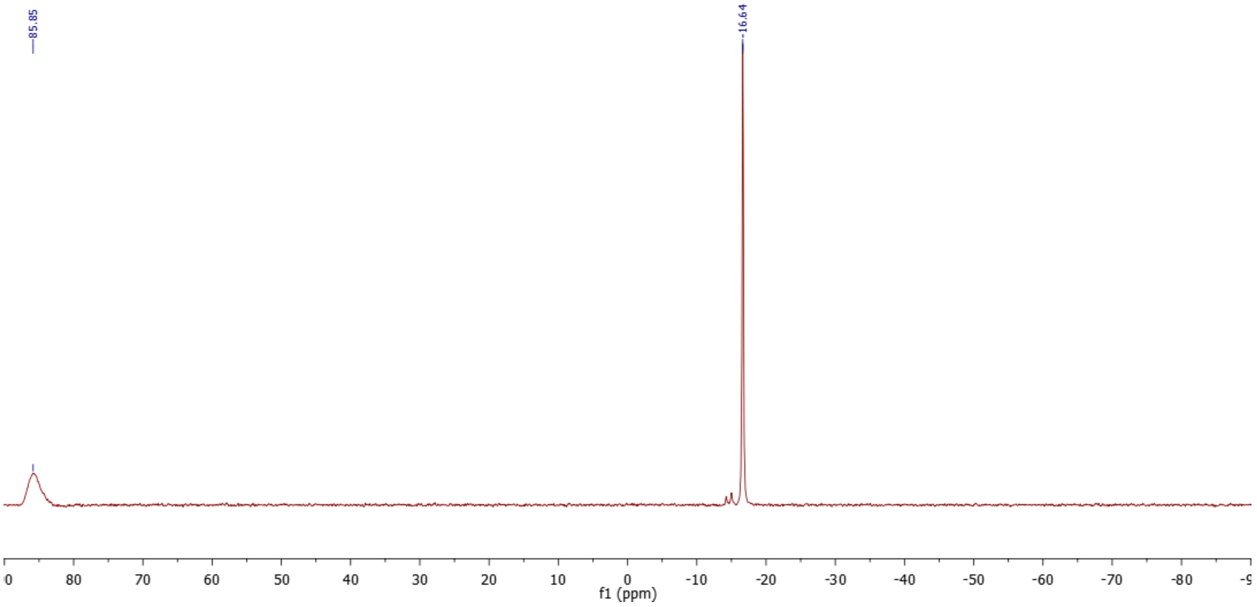

^{13}C NMR (101 MHz, CD_2Cl_2, 298K):

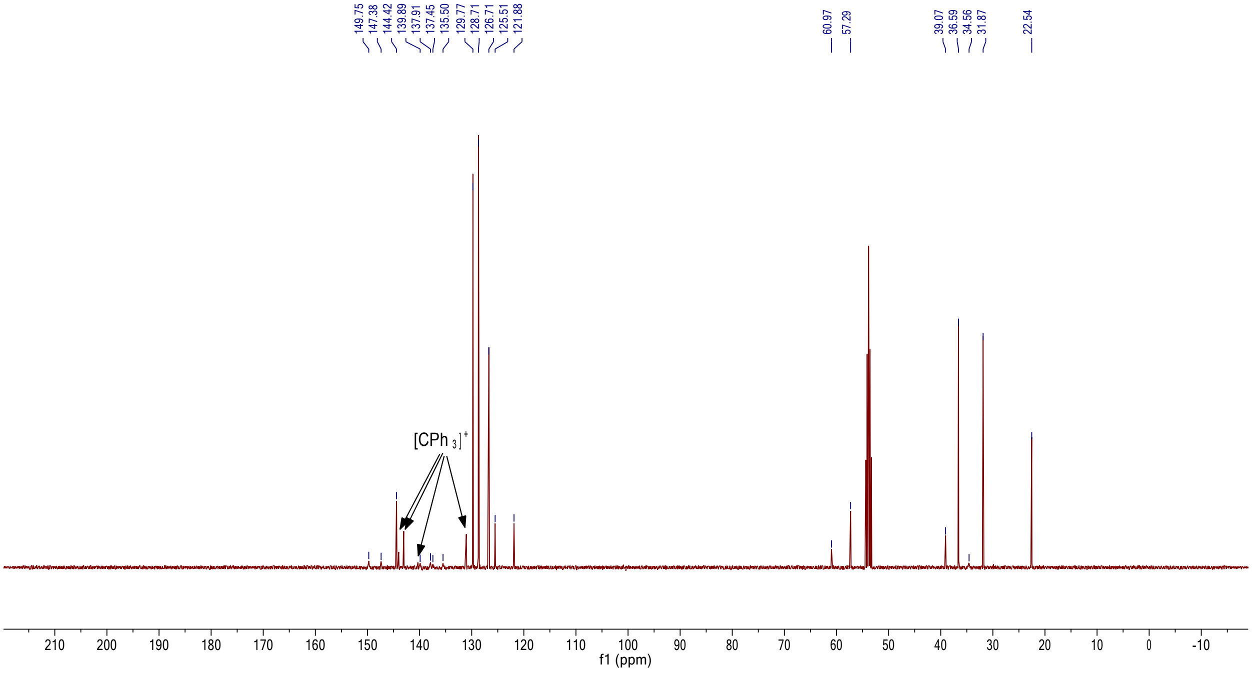

^{19}F NMR (376 MHz, CD_2Cl_2, 298K):

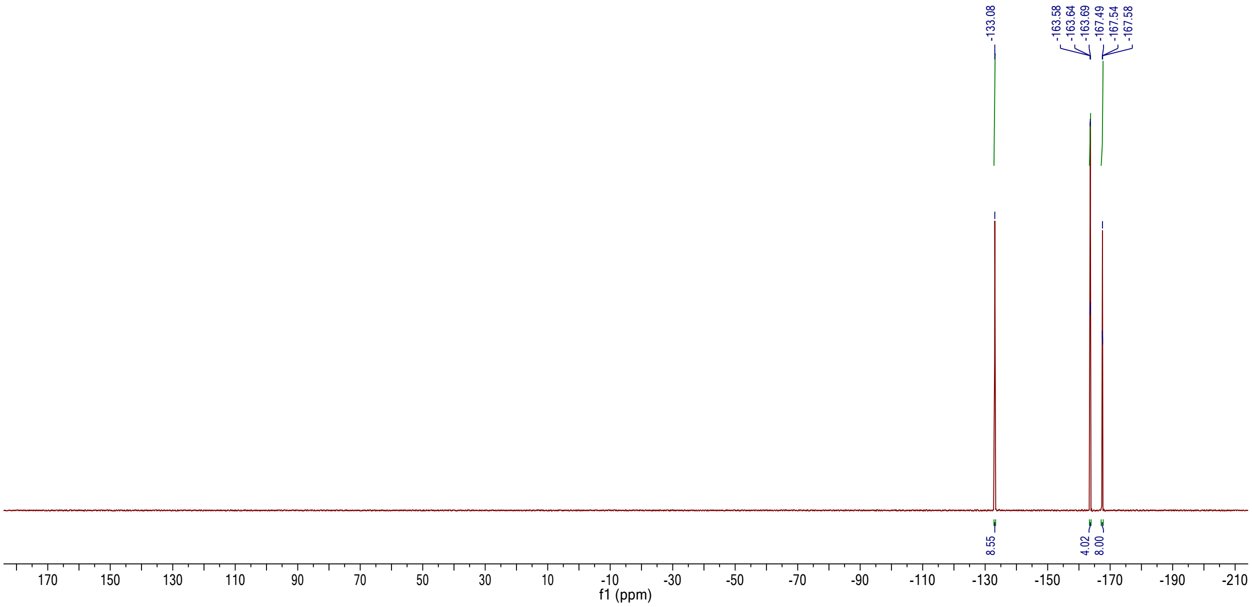

[1-methyl-3-phenylimidazol-2-ylidene-9-borabicyclo[3.3.1]nonane][tetrakis(pentafluorophenyl)borate] [(PhIMe)(BC_8H_{14})][$B(C_6F_5)_4$], (7b).

^{1}H NMR (500 MHz, CD_2Cl_2, 298K, $HCPh_3$ omitted): δ 7.71-7.67 (m, 1H), 7.65-6.60 (m, 2H), 7.61 (d, 1H, $^3J_{HH}$ = 1.8 Hz), 7.50 (d, 1H, $^3J_{HH}$ = 1.8 Hz), 7.37-7.33 (m, 2H), 4.11 (s, 3H), 2.11-1.98 (m, br, 6H), 1.73 (br, 2H), 1.69-1.59 (m, br, 4H), 1.44-1.35 (m, br, 2H). ^{11}B NMR (128 MHz, CD_2Cl_2, 298K): δ 82.47, -16.66. ^{13}C NMR (126 MHz, CD_2Cl_2, 298K, partial, $HCPh_3$ omitted): δ 148.6 (dm, $^1J_{CF}$ = 243 Hz), 138.7 (dm, $^1J_{CF}$ = 242 Hz), 136.7 (dm, $^1J_{CF}$ = 244 Hz), 135.9, 132.4, 131.0, 125.0, 126.8, 126.3, 38.0, 34.8, 33.6 (br), 22.7. ^{19}F NMR (377 MHz, 298K, CD_2Cl_2): δ -133.1 (m, 8F, *o*–C_6F_5), -163.6 (t, 4F, $^3J_{FF}$ = 20 Hz, *p*–C_6F_5), -167.5 (t, 8F, $^3J_{FF}$ = 18 Hz, *m*–C_6F_5).

^{1}H NMR (500 MHz, CD_2Cl_2, 298K):

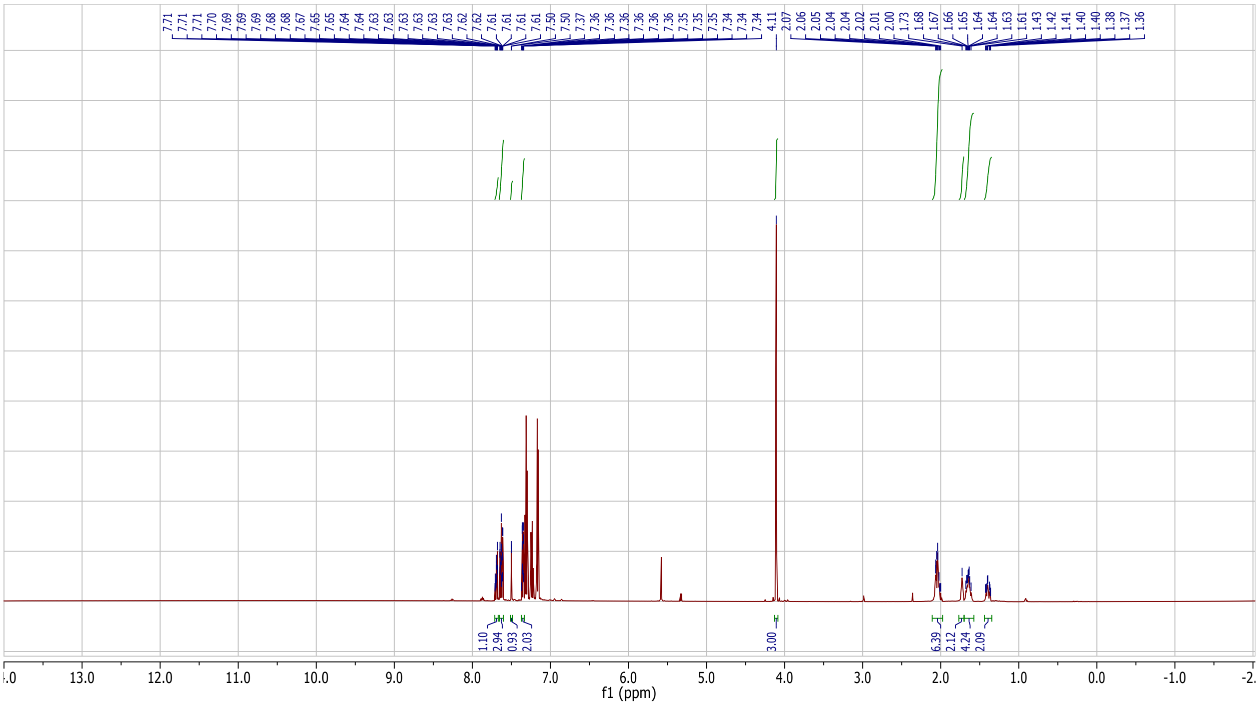

^{11}B NMR (128 MHz, CD_2Cl_2, 298K):

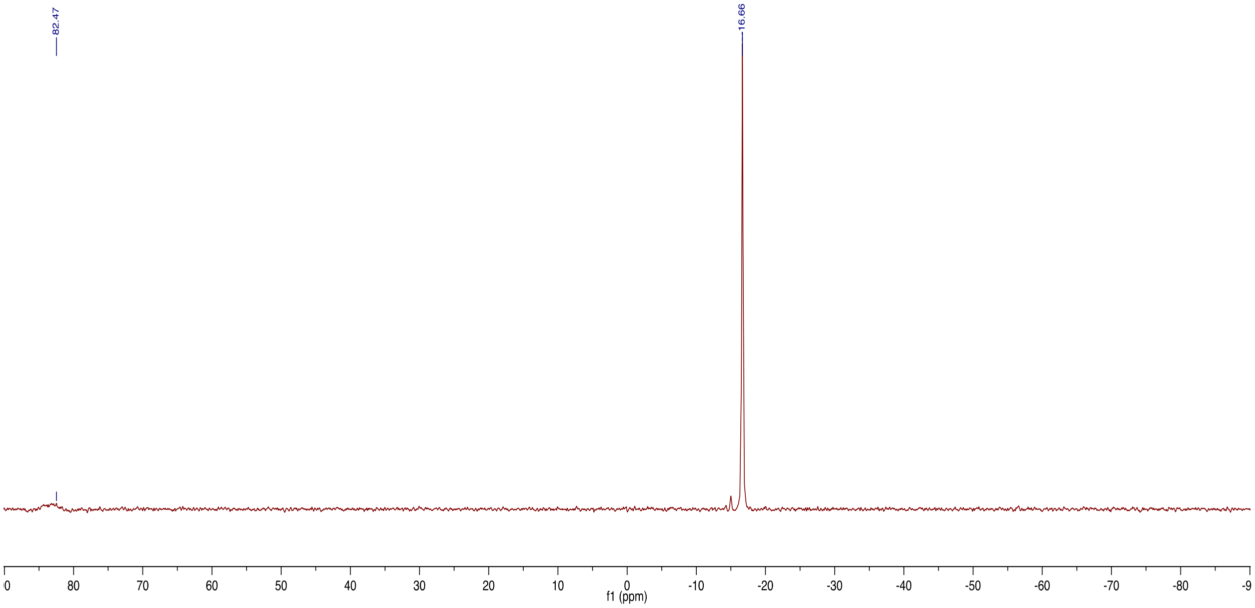

^{13}C NMR (126 MHz, CD_2Cl_2, 298K):

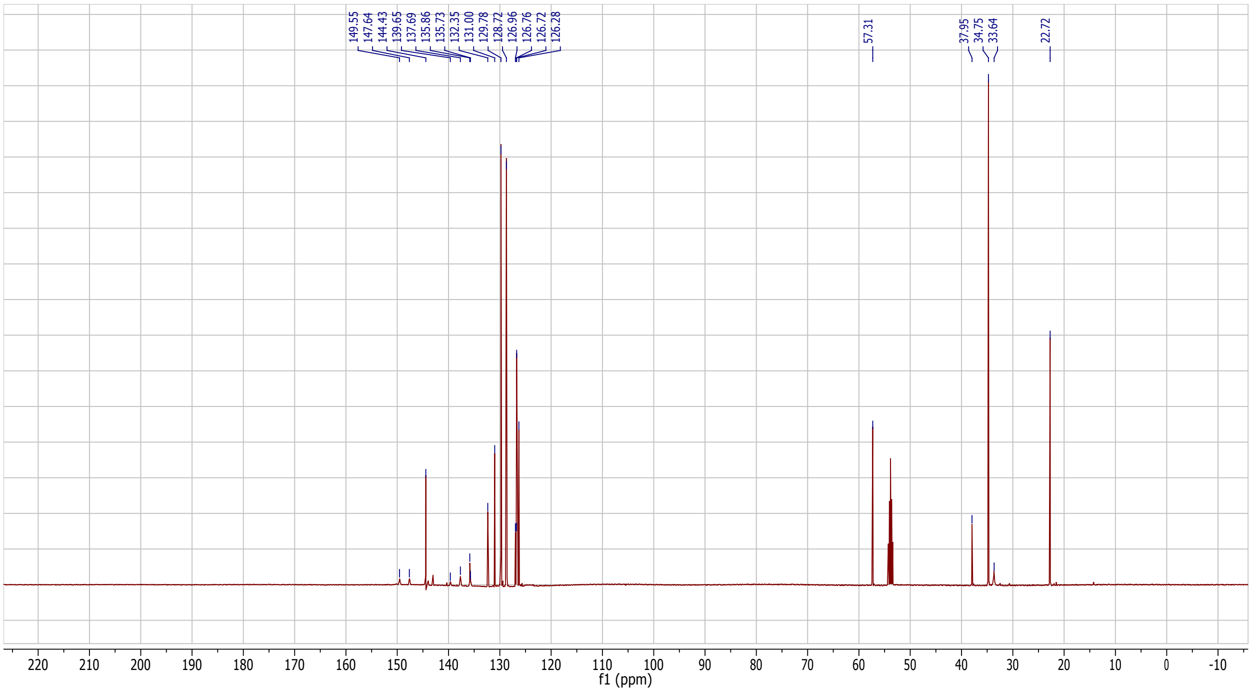

^{19}F NMR (377 MHz, 298K, CD_2Cl_2):

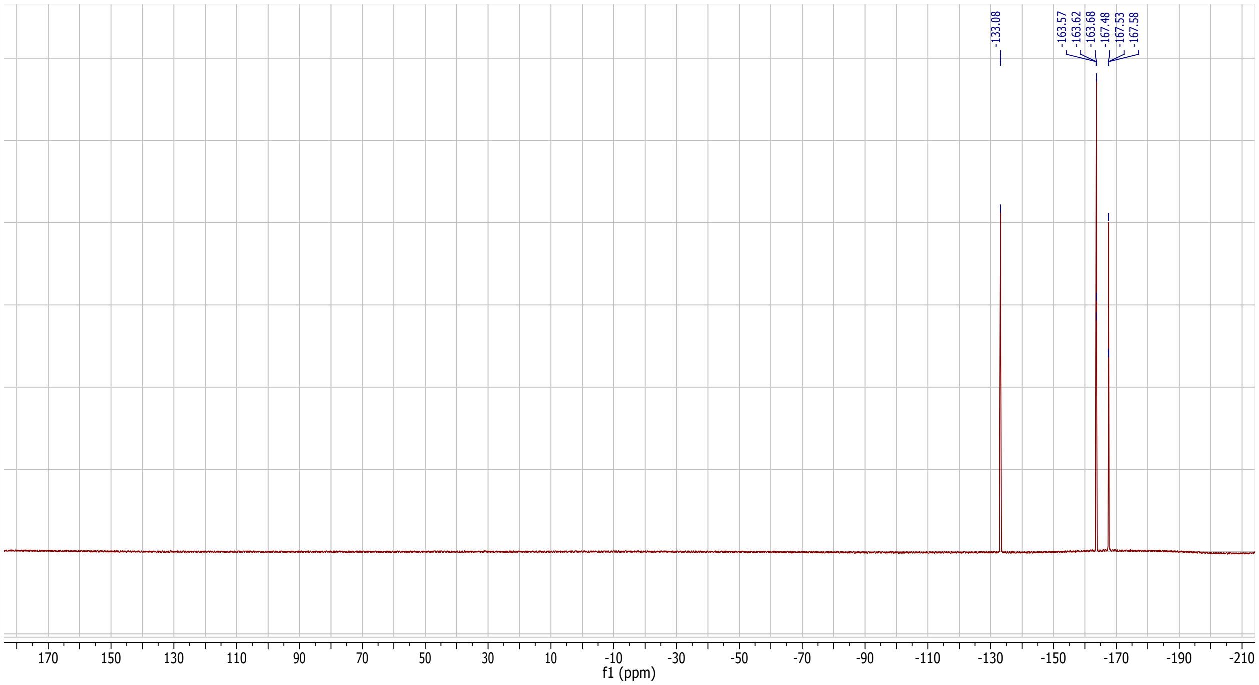

[1,3-dimethylimidazol-2-ylidene-9-borabicyclo[3.3.1]nonane][tetrakis(pentafluorophenyl)borate] [$(IMe_2)(BC_8H_{14})][B(C_6F_5)_4]$ (8b).

^{1}H NMR (500 MHz, CD_2Cl_2, 298 K, $HCPh_3$ omitted): δ 7.37 (s, 2H), 3.98 (s, 6H), 2.30-2.10 (m, 8H), 2.00-1.90 (m, 4H), 1.54-1.45 (m, 2H). ^{11}B NMR (128 MHz, CD_2Cl_2): δ 82.70, -16.67. ^{13}C NMR (126 MHz, CD_2Cl_2, 298 K, partial, $HCPh_3$ omitted): δ 148.6 (dm, $^{1}J_{CF}$ = 240 Hz), 138.7 (dm, $^{1}J_{CF}$ = 243 Hz), 136.7 (dm, $^{1}J_{CF}$ = 242 Hz), 127.0, 38.0, 34.4, 33.1 (br), 22.9.^{19}F NMR (377 MHz, CD_2Cl_2, 298 K): δ –133.1 (m, br, 8F, *o*–C_6F_5), –163.6 (t, 4F, *p*–C_6F_5, $^{3}J_{FF}$ = 20.5 Hz), –167.6 (t, br, 8F, *m*–C_6F_5, $^{3}J_{FF}$ = 18 Hz).

^{1}H NMR (500 MHz, CD_2Cl_2, 298 K):

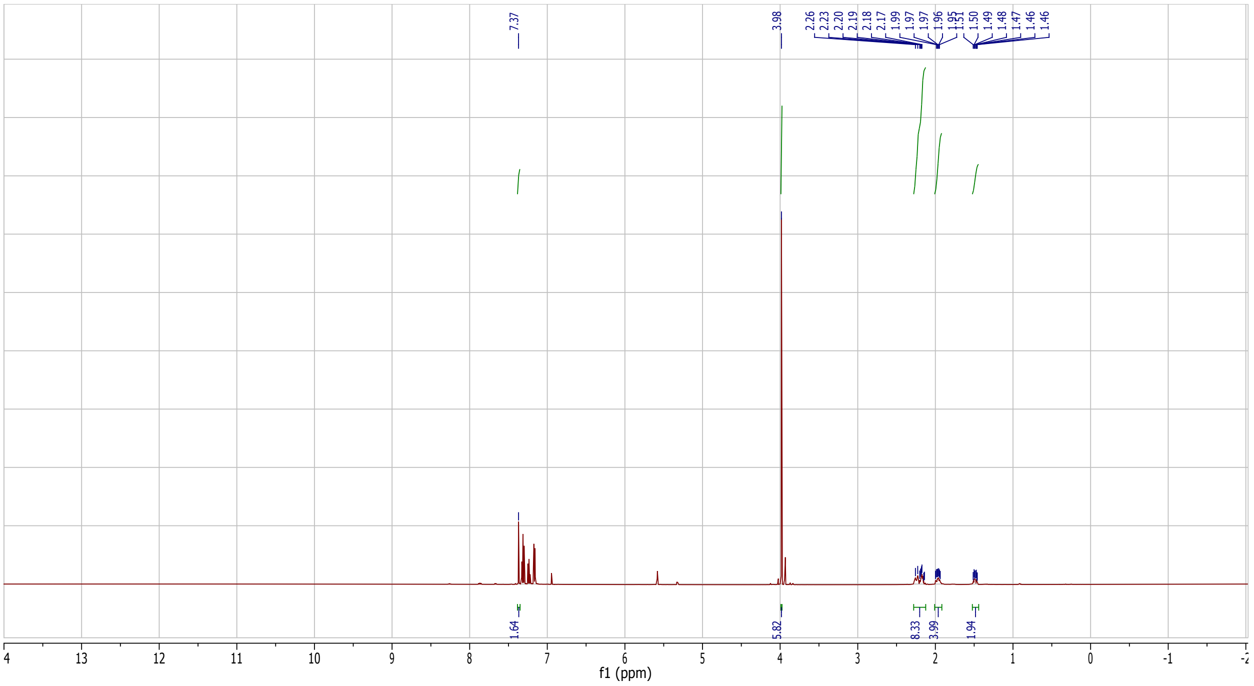

^{11}B NMR (128 MHz, CD_2Cl_2):

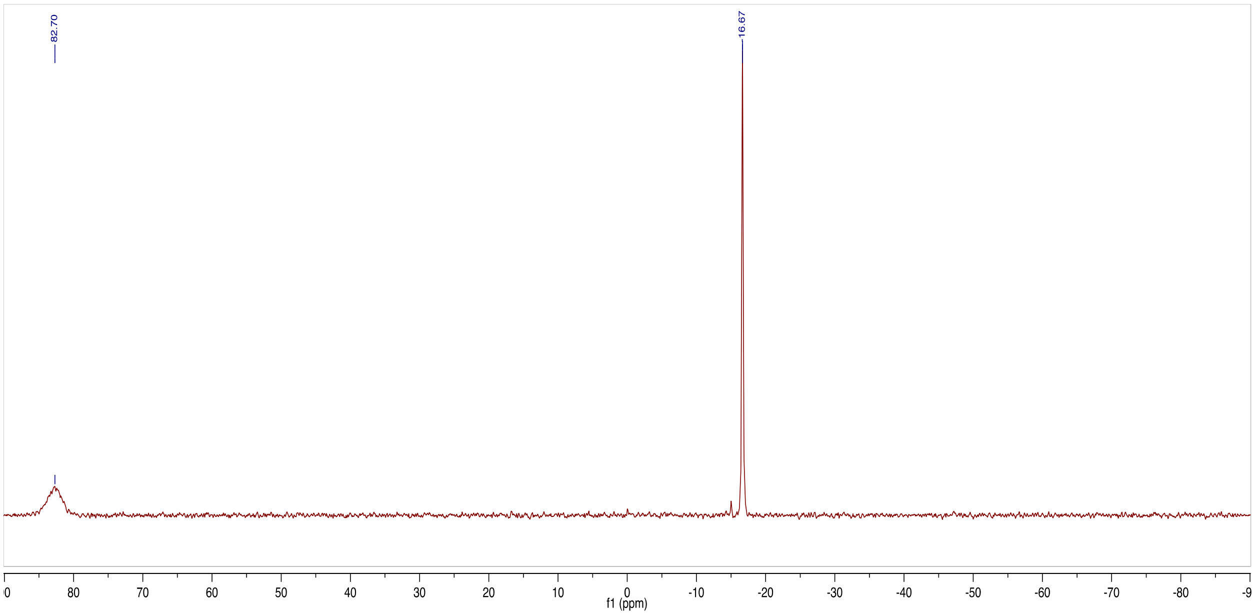

^{13}C NMR (126 MHz, CD_2Cl_2, 298 K):

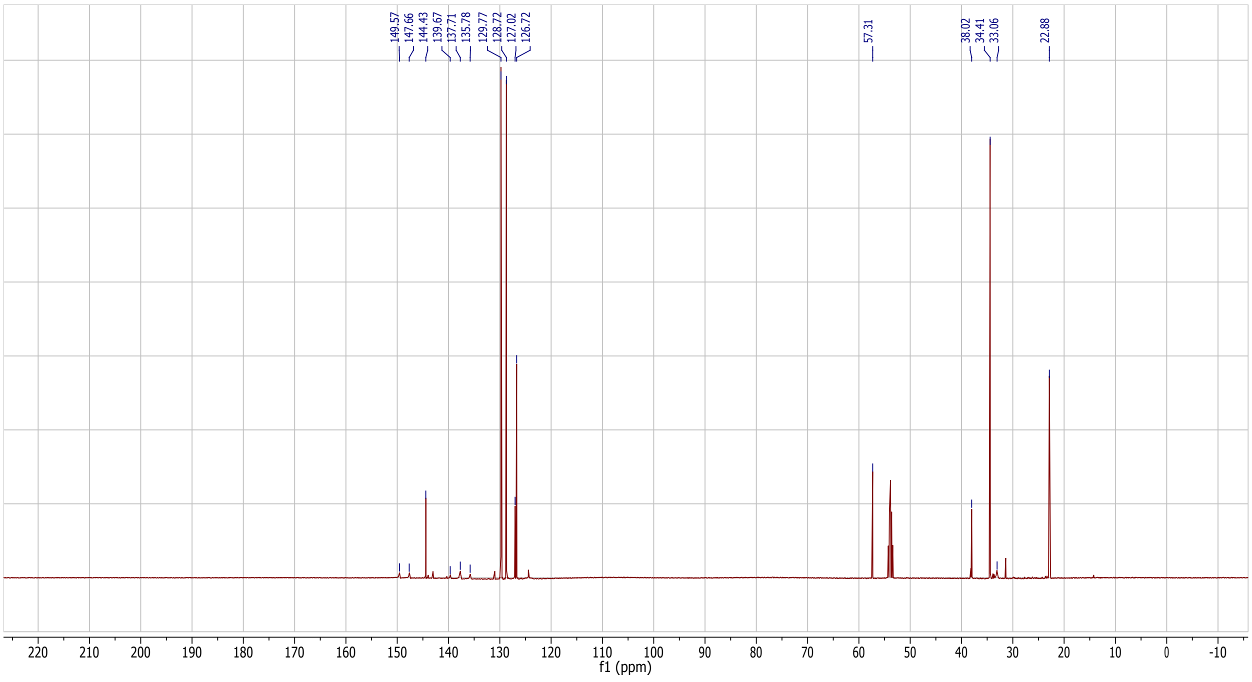

^{19}F NMR (377 MHz, CD_2Cl_2, 298 K):

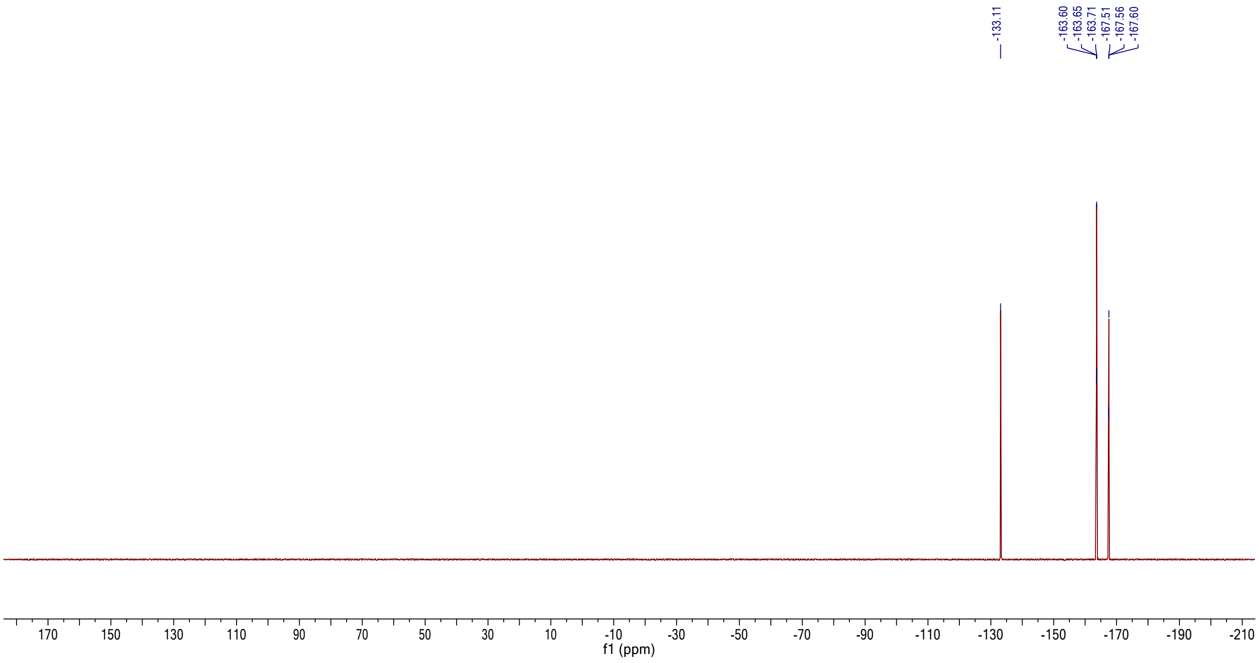

[1,3,4,5-tetramethylimidazol-2-ylidene-9-borabicyclo[3.3.1]nonane][tetrakis(pentafluorophenyl)borate] $[(IMe_4)(HBC_8H_{14})][B(C_6F_5)_4]$, (9b).

^{1}H NMR (400 MHz, CD_2Cl_2, 298 K, $HCPh_3$ omitted): δ 3.80 (s, 6H), 2.31 (s, 6H), 2.28-2.05 (m, 8H), 2.02-1.86 (m, 4H), 1.55-1.43 (m, br, 2H). ^{11}B NMR (CD_2Cl_2, 128 MHz): δ 81.5 (br), –16.84 (s). ^{13}C{^{1}H} NMR (101 MHz, CD_2Cl_2, 298 K, partial, $HCPh_3$ omitted): δ 148.6 (dm, $^1J_{CF}$ = 244 Hz), 138.7 (dm, $^1J_{CF}$ = 241 Hz); 136.7.8 (dm, $^1J_{CF}$ = 250 Hz), 132.1, 34.5, 34.4, 33.2 (br), 22.9, 9.1. ^{19}F NMR (CD_2Cl_2, 376 MHz): δ –133.1 (m, br, 8F, *o*–C_6F_5), –163.6 (t, 4F, *p*–C_6F_5, $^3J_{FF}$ = 20 Hz); –167.6 (t, br, 8F, *m*–C_6F_5, $^3J_{FF}$ = 18 Hz).

^{1}H NMR (400 MHz, CD_2Cl_2, 298 K):

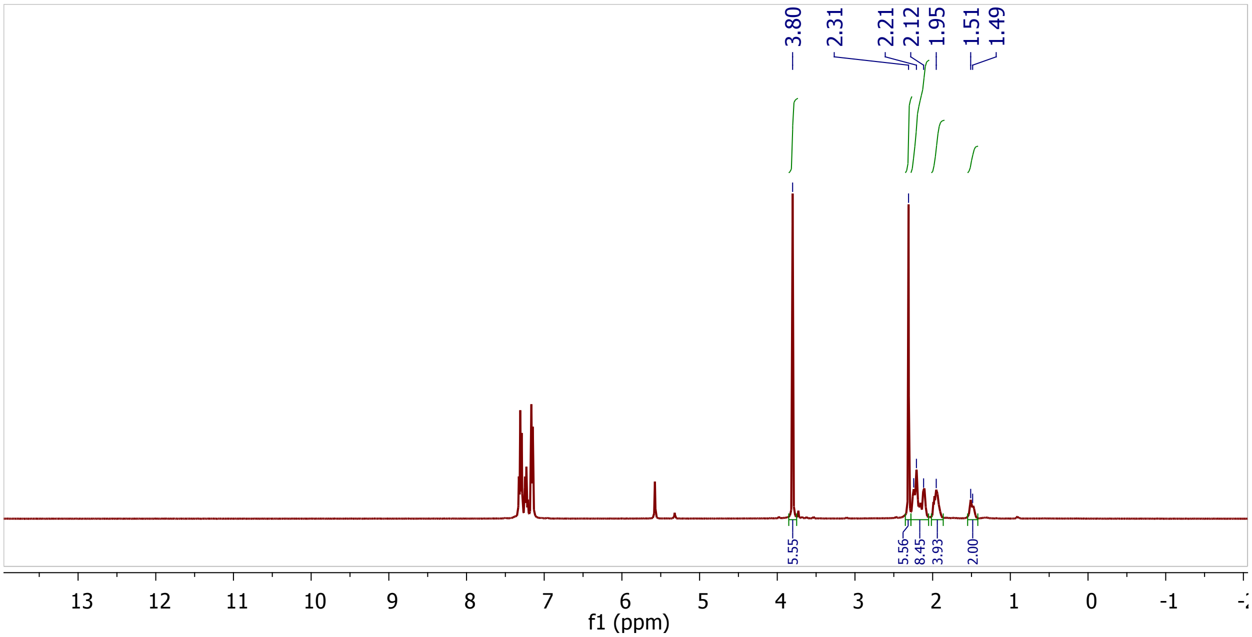

^{11}B NMR (CD_2Cl_2, 128 MHz, 298 K):

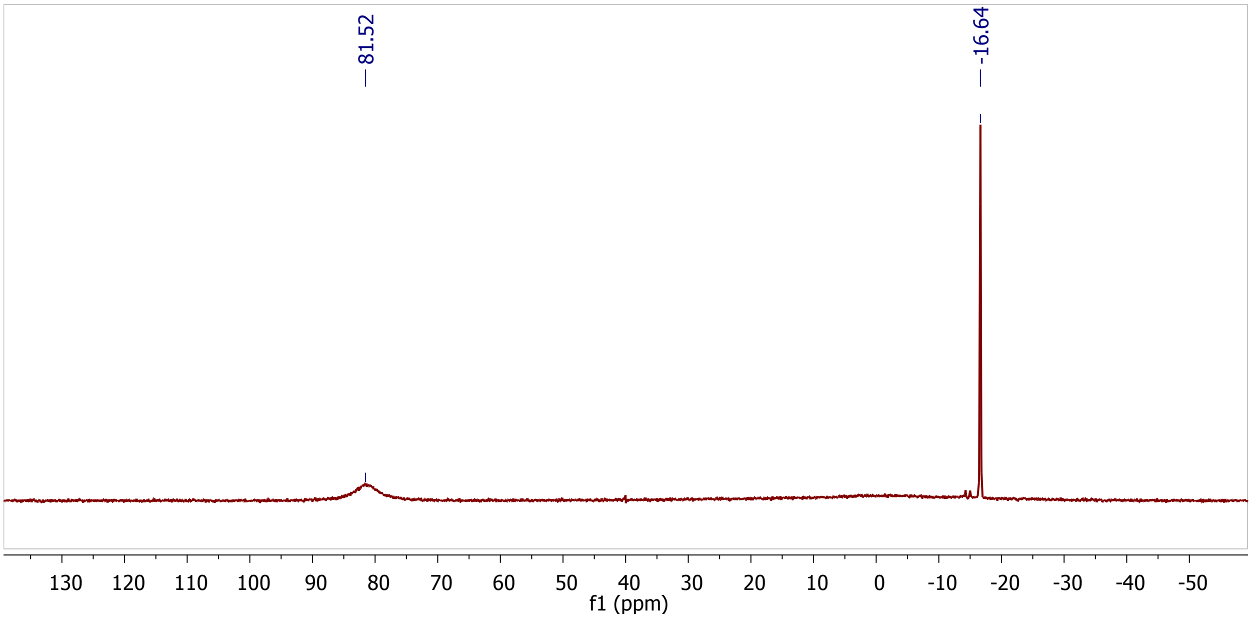

$^{13}C\{^1H\}$ NMR (101 MHz, CD_2Cl_2, 298 K):

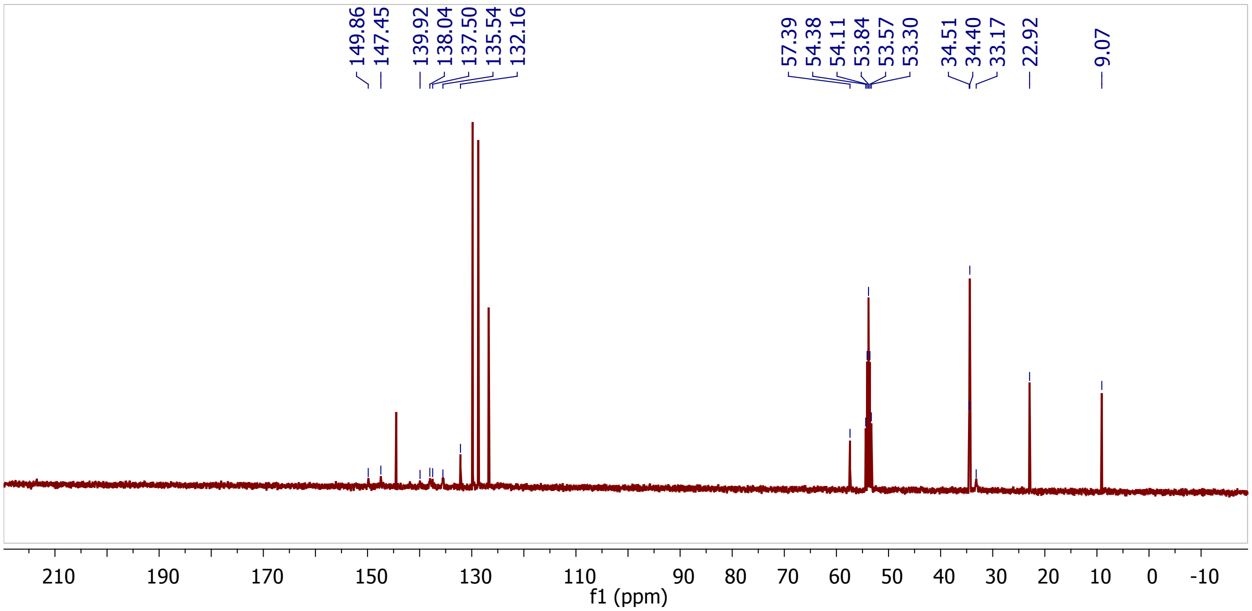

^{19}F NMR (CD_2Cl_2, 376 MHz, 298 K):

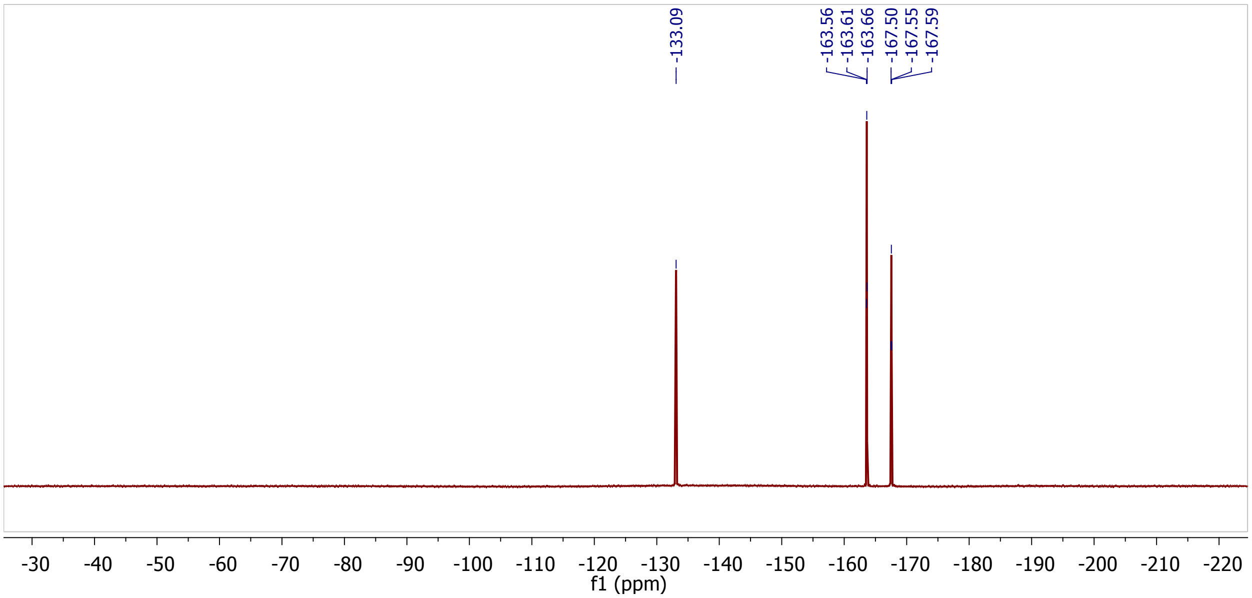

[4,5-dichloro-1,3-dimethylimidazol-2-ylidene-9-borabicyclo[3.3.1]nonane][tetrakis(pentafluorophenyl)borate] [(Cl_2Me_2I)(BC_8H_{14})][B(C_6F_5)$_4$], (10b).

^{1}H, ^{13}C, ^{11}B and ^{19}F NMR spectra were consistent with those for isolated **10b** reported herein.

[4,5-dichloro-1,3-dimethylimidazol-2-ylidene-9-borabicyclo[3.3.1]nonane][tetrakis(pentafluorophenyl)borate] [(Cl_2Me_2I)(BC_8H_{14})][B(C_6F_5)$_4$], (10b).

In an inert atmosphere glovebox, **10a** (126.4 mg, 0.4404 mmol) was dissolved in 3 mL dichloromethane. A solution of [Ph_3C][B(C_6F_5)$_4$] (406.2 mg, 0.4404 mmol) in 2 mL dichloromethane was added dropwise to the borane solution. The reaction mixture was layered with pentane and cooled to -35˚C to give [(Cl_2Me_2I)(BC_8H_{14})][B(C_6F_5)$_4$], **10b**, as colorless crystals. After washing with 3 x 5 mL pentane and drying *in vacuo* 390.0 mg **10b** was collected

as a white solid. ^{1}H NMR (400 MHz, CD_2Cl_2, 298 K): δ 3.86 (s, 6H), 2.25-1.75 (m, br, 8H), 1.90-1.75 (m, br, 4H), 1.45-1.33 (m, br, 2H). ^{11}B NMR (CD_2Cl_2, 128 MHz, 298 K): δ 82.88 (br), –16.66 (s). ^{13}C{^{1}H} NMR (101 MHz, CD_2Cl_2, 298 K, partial): δ 148.5 (dm, 8C, $^1J_{CF}$ = 240 Hz), 138.6 (dm, 4C, $^1J_{CF}$ = 241 Hz); 136.7 (dm, 8C, $^1J_{CF}$ = 244 Hz), 36.2, 34.7, 33.8 (br), 22.8. ^{19}F NMR (CD_2Cl_2, 376 MHz, 298 K): δ –133.1 (m, br, 8F, *o*–C_6F_5); –163.6 (t, 4F, *p*–C_6F_5, $^3J_{FF}$ = 20 Hz); –167.6 (t, br, 8F, *m*–C_6F_5, $^3J_{FF}$ = 19 Hz). Anal. Calcd. for $C_{37}H_{20}B_2N_2F_{20}Cl_2$: C 46.05%, H 2.09%, N 2.90%. Found: C 46.14%, H 1.58%, N 3.11%.

Synthesis of trimethylphosphine-9-borabicyclo[3.3.1]borane (11).

In an inert atmosphere glovebox, 2 mL of 1M PMe_3 in toluene was added dropwise to a stirring suspension of 9-BBN dimer (244 mg, 1.00 mmol) in 5 mL toluene. The reaction was stirred overnight and recrystallized at -35ºC to give colourless crystals. The supernatant was decanted and the crystals were washed with 3 x 2 mL cold toluene followed by 3 x 2 mL cold pentane and subsequently dried *in vacuo* to give Me_3P·9-BBN (**11**) as a white crystalline solid (325 mg, 82.1 %yield). ^{1}H NMR (400 MHz, CD_2Cl_2, 298 K): δ 2.02-1.42 (m, 12H), 1.29 (d, 9H, $^2J_{PH}$ = 9.6 Hz), 0.96 (br, 2H). ^{11}B NMR (128 MHz, CD_2Cl_2, 298 K): δ –14.92 (dd, $^1J_{BH}$ = 88 Hz, $^1J_{BP}$ = 48 Hz). ^{31}P{^{1}H} NMR (162 MHz, CD_2Cl_2, 298 K): –13.0 ($^1J_{BP}$ = 45 Hz) ^{13}C{^{1}H} NMR (101 MHz, CD_2Cl_2, 298 K): 36.7, 36.6, 33.15, 33.12, 26.2, 25.5, 20.7 (br), 11.8 ($^1J_{PC}$ = 32.5 Hz). HRMS (DART-EI+): mass [M-H] calc'd for $^{12}C_{11}{}^1H_{23}{}^{11}B^{31}P$: 197.16304, found: 197.16311. Anal. Calcd. for $C_{11}H_{24}BP$: C 66.69%, H 12.21%, N 0.00%. Found: C 66.48%, H 12.71%, N 0.13%.

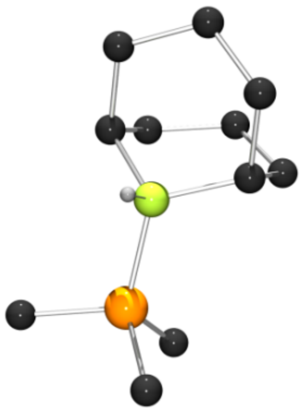

POV-ray depiction of 11. C: black, B: yellow-green, N: blue, H: grey. Some H-atoms omitted for clarity.

Synthesis of N-(*p*-methoxycarbonyl)benzyl-N-*tert*-butylamine.

In an inert atmosphere glovebox, methyl 4-formylbenzoate (10.0 mmol, 1.642 g) was weighed into a round-bottom flask and dissolved in 50 mL diethyl ether. Activated 4Å molecular sieves (6 g) and *tert*-butylamine (0.872 g, 12.0 mmol) were added to the reaction vessel and reacted for 5 days at room temperature. Volatiles were removed *in vacuo* and the residue was extracted with CH_2Cl_2 (5 x 8mL) and concentrated *in vacuo*. The resulting white solid was recrystallized from pentane and dried *in vacuo* to afford 0.9195 g N-(*p*-methoxycarbonyl)benzyl-N-*tert*-butylamine as a white solid (42% yield). ^{1}H NMR (400 MHz, CD_2Cl_2): δ 8.34 (s, 1H), 8.11 (d, 2H, $^3J_{HH}$= 8.0 Hz), 7.87 (d, 2H, $^3J_{HH}$= 8.0 Hz), 3.94 (s, 3H), 1.37 (s, 9H). ^{13}C NMR (101 MHz, CD_2Cl_2): δ 167.0, 154.4, 141.7, 131.9, 130.0, 128.1, 58.0, 52.4, 29.7. HRMS (DART-EI+): mass [M+H] calc'd for $^{12}C_{13}{}^1H_{18}{}^{14}N_1{}^{16}O_2$: 220.13375, found: 220.13431. Anal. Calcd. for $C_{13}H_{17}NO_2$: C 71.21%, H 7.81%, N 6.39% Found: C 71.16%, H 8.14%, N 6.51%.

Procedures for Elevated Pressure Reductions.

Procedure 1 (*in situ*), (Table 1, Entries 1-10): In an inert atmosphere glovebox, NHC-borenium salts **4b** – **10b** were generated *in situ* at appropriate catalyst loadings using the protocols described above with 0.4 mL dichloromethane used in place of deuterated solvent. The NHC-borenium salt solution was transferred to a vial containing N-benzylidene-*tert*-butylamine (1.824 mmol, 294.1 mg) using a further 0.2 mL dichloromethane. This vial was equipped with a stir bar and placed in a Parr pressure reactor. The reactor was sealed, removed from the glovebox and attached to a thoroughly purged hydrogen gas line. The reactor was purged ten times at 50 atm with hydrogen gas and ten times at 102 atm with hydrogen gas. The reactor was sealed under 102 atm hydrogen gas and stirred magnetically for the indicated time at room temperature. The reactor was slowly vented and an NMR sample of the reaction mixture was taken in $CDCl_3$. Conversion of unsaturated substrate to amine product was determined by ^1H NMR spectroscopy.

Procedure 2 (isolated catalyst), (Table 1, Entries 11-13 and Table 2): In an inert atmosphere glovebox, an unsaturated substrate (1.824 mmol (Table 1, Entries 11 and 12) or 1.000 mmol (Table 1, Entry 13), or 0.500 mmol (Table 2)) and an appropriate loading of **10b** were weighed into vials. **10b** was transferred to the vial containing the substrate with 0.6 mL (Table 1, Entries 11, 12 and 14), 0.4 mL (Table 2, Entries 2-6) or 0.3 mL (Table 1, Entry 13 and Table 2, Entries 1, 7-10) CH_2Cl_2. The reaction vial was equipped with a stir bar and placed in a Parr pressure reactor. The reactor was sealed, removed from the glovebox and attached to a thoroughly purged hydrogen gas line. The reactor was purged ten times at 50 atm with hydrogen gas and ten times at 102 atm with hydrogen gas. The reactor was sealed under 102 atm hydrogen gas and stirred

magnetically for the indicated time at room temperature. The reactor was slowly vented and an NMR sample of the reaction mixture was taken in $CDCl_3$. Conversion of unsaturated substrate to amine product was determined by ^{1}H NMR spectroscopy.

Conversion to N-benzyl-1,1-diphenylmethanamine[19] was assessed by comparison to literature ^{1}H-NMR values. Where isolated yields are reported, the NMR sample was recombined with the reaction mixture and concentrated to dryness *in vacuo*. The residue was extracted with 19:1 hexanes : ethyl acetate, filtered through a plug of silica pre-treated with diethylamine and concentrated *in vacuo* to give hydrogenated products.

N-benzyl(*tert*-butylamine):[20]

Colourless oil, 134 mg, 83% yield. ^{1}H NMR ($CDCl_3$, 500 MHz, 298K): δ 7.28-7.20 (m, 4H), 7.14 (tm, 1H, $^{3}J_{HH}$ = 7.2 Hz), 3.65 (s, 2H), 1.11 (s, 9H), N-*H* not observed. ^{13}C NMR (126 MHz, $CDCl_3$, 298K): δ 141.5, 128.4, 128.3, 126.7, 50.7, 47.3, 29.2. HRMS (DART-EI+): mass [M+H] calc'd for $^{12}C_{11}{}^{1}H_{17}{}^{14}N_1$: 164.14392, found: 164.14442.

^{1}H NMR ($CDCl_3$, 500 MHz, 298K):

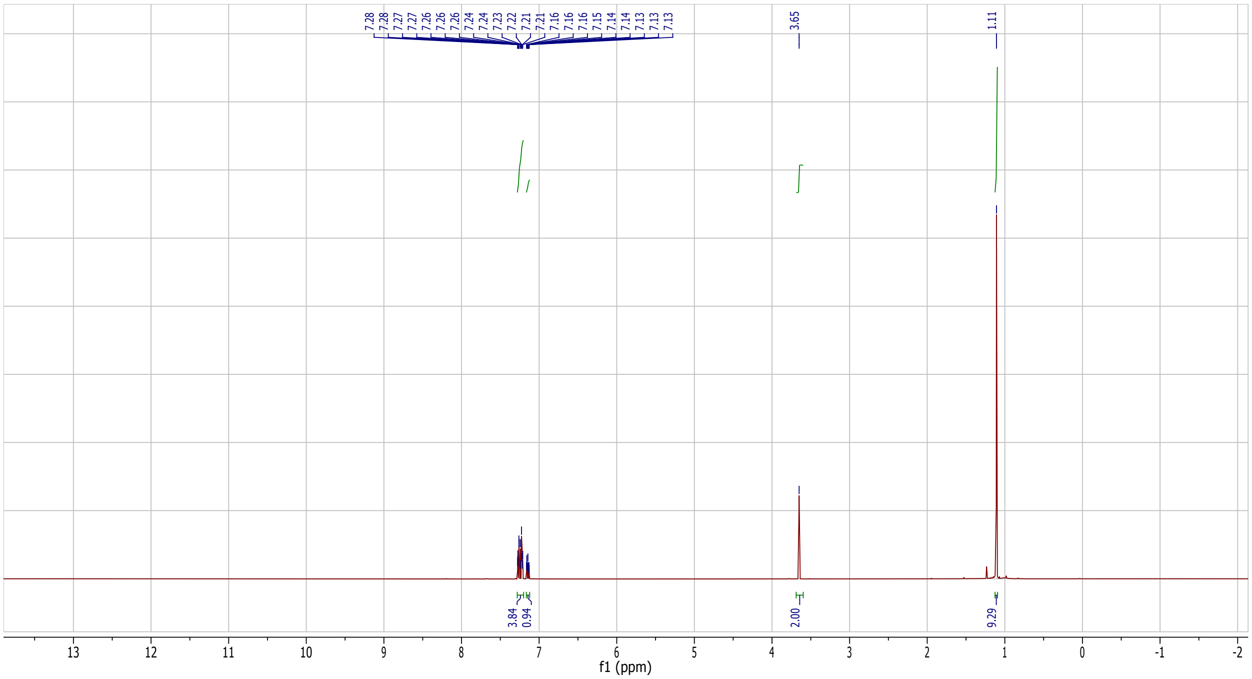

^{13}C NMR (126 MHz, $CDCl_3$, 298K):

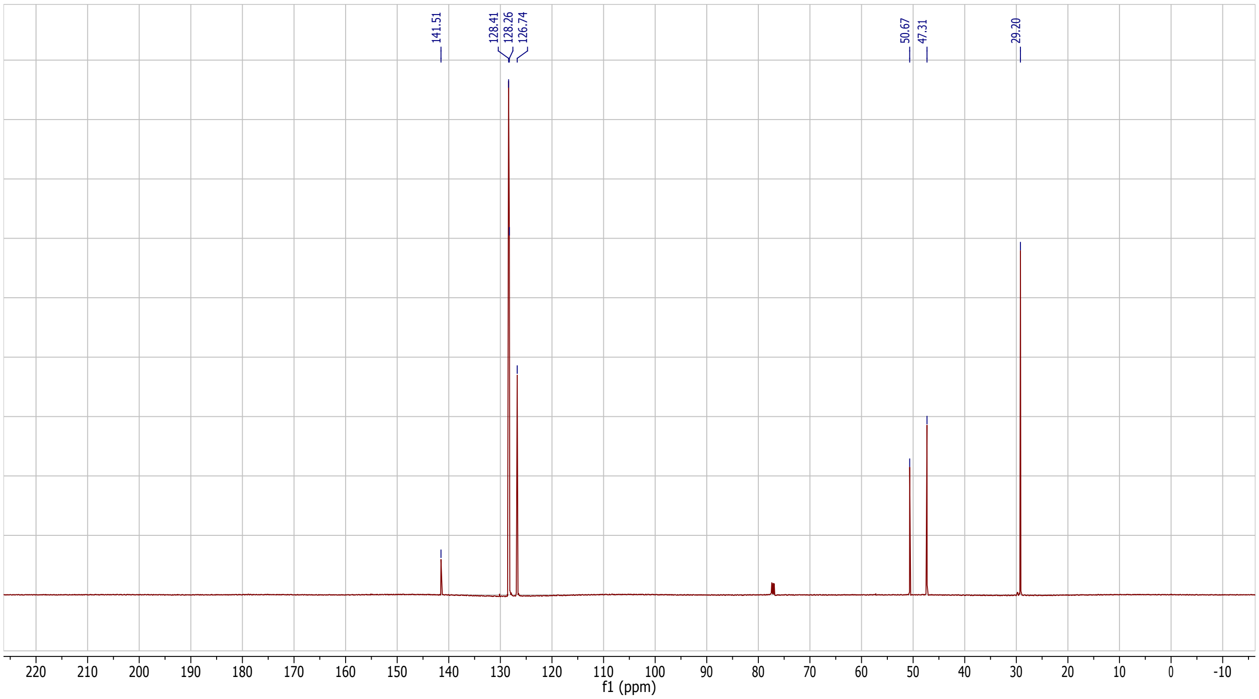

N-(*p*-methoxycarbonyl)benzyl-N-*tert*-butylamine:[21]

Colourless oil, 91 mg, 82% yield. ^{1}H NMR ($CDCl_3$, 500 MHz, 298K): δ 7.97 (d, 2H, $^{3}J_{HH}$ = 7.8 Hz), 7.40 (d, 2H, $^{3}J_{HH}$ = 7.8 Hz), 3.88 (s, 3H), 3.77 (s, 2H), 1.16 (s, 9H), N-*H* not observed. ^{13}C NMR (126 MHz, $CDCl_3$, 298K): δ 167.2, 147.1, 129.8, 128.7, 128.2, 52.1, 50.9, 47.1, 29.2. HRMS (DART-EI+): mass [M+H] calc'd for $^{12}C_{13}{}^{1}H_{20}{}^{14}N_{1}{}^{16}O_{2}$: 222.14940, found: 222.15044.

^{1}H NMR ($CDCl_3$, 500 MHz, 298K):

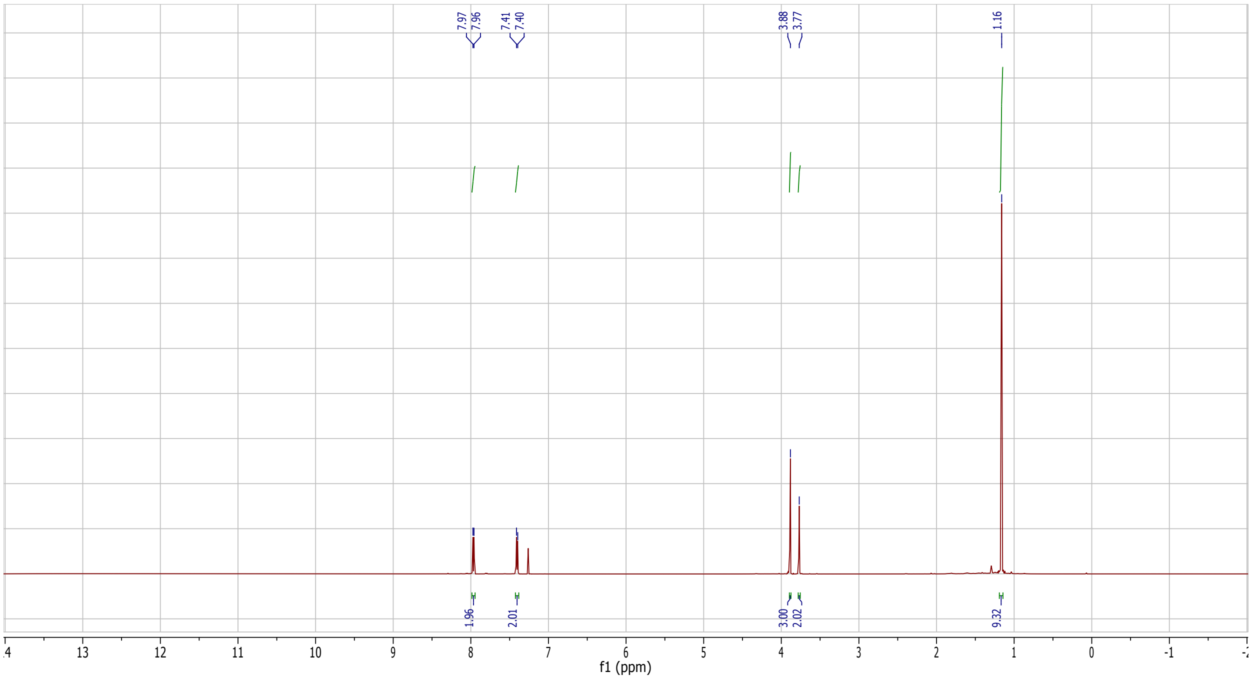

^{13}C NMR (126 MHz, $CDCl_3$, 298K):

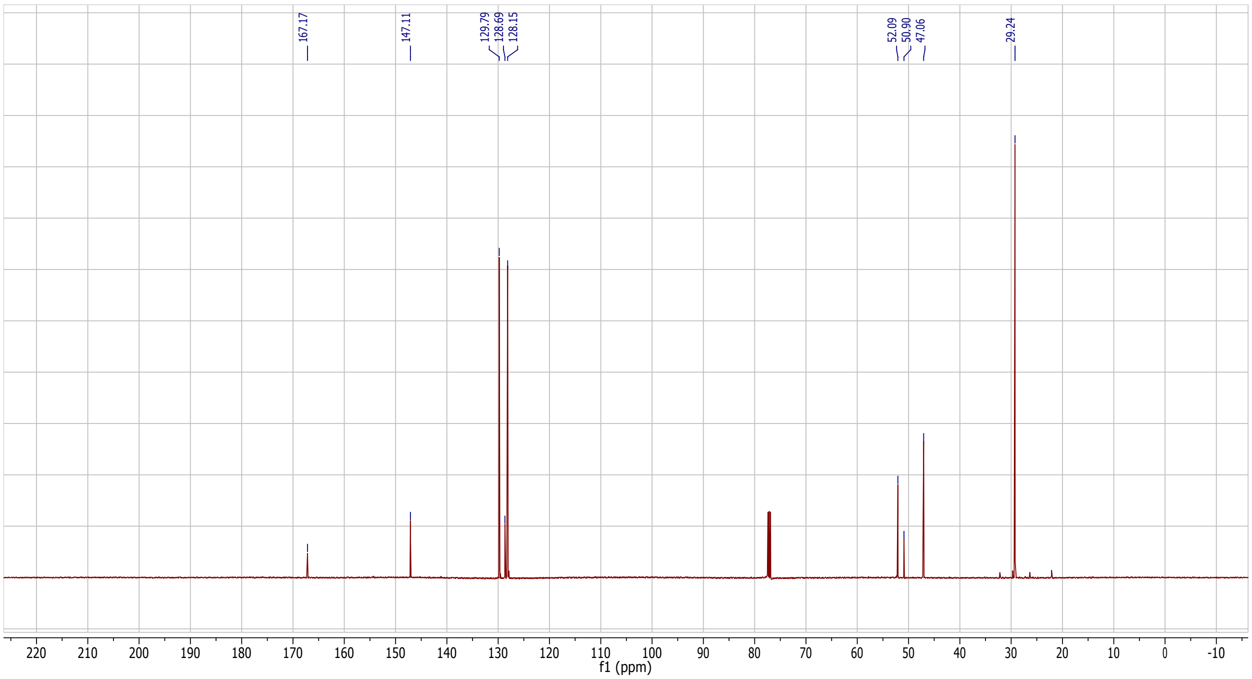

N-*o*-chlorobenzyl(*tert*-butylamine):[10]

Colourless oil, 97 mg, 98% yield. ^{1}H NMR ($CDCl_3$, 500 MHz, 298K): δ 7.45 (dm, 1H, $^{3}J_{HH}$ = 7.6 Hz), 7.33 (dd, 1H, $^{3}J_{HH}$ = 7.8 Hz, $^{4}J_{HH}$ = 1.4 Hz), 7.22 (td, 1H, $^{3}J_{HH}$ = 7.5 Hz, $^{4}J_{HH}$ = 1.4 Hz), 7.17 (td, 1H, $^{3}J_{HH}$ = 7.5 Hz, $^{4}J_{HH}$ = 1.8 Hz), 3.82 (s, 2H), 1.20 (s, 9H), N-*H* not observed. ^{13}C NMR (126 MHz, $CDCl_3$, 298K): δ 138.8, 133.8, 130.5, 129.5, 128.2, 127.1, 51.0, 45.0, 29.2. HRMS (DART-EI+): mass [M+H] calc'd for $^{12}C_{11}{}^{1}H_{17}{}^{35}Cl_{1}{}^{14}N_{1}$: 198.10495, found: 198.10536.

^{1}H NMR ($CDCl_3$, 500 MHz, 298K):

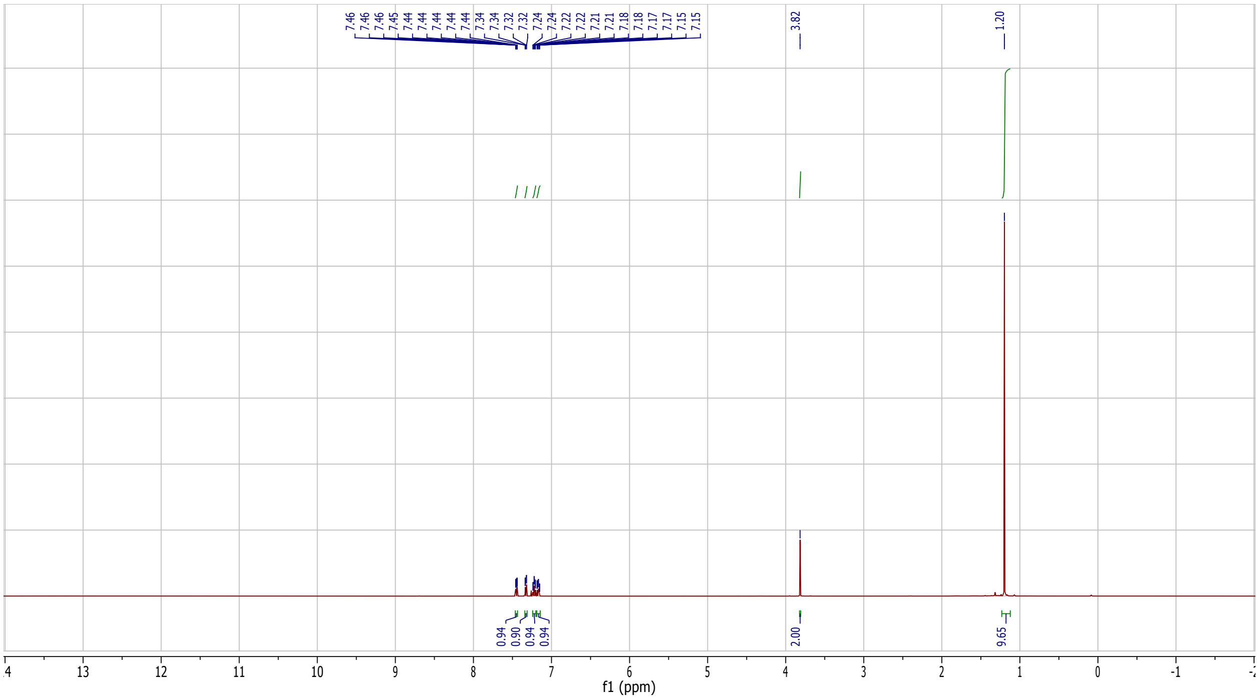

^{13}C NMR (126 MHz, $CDCl_3$, 298K):

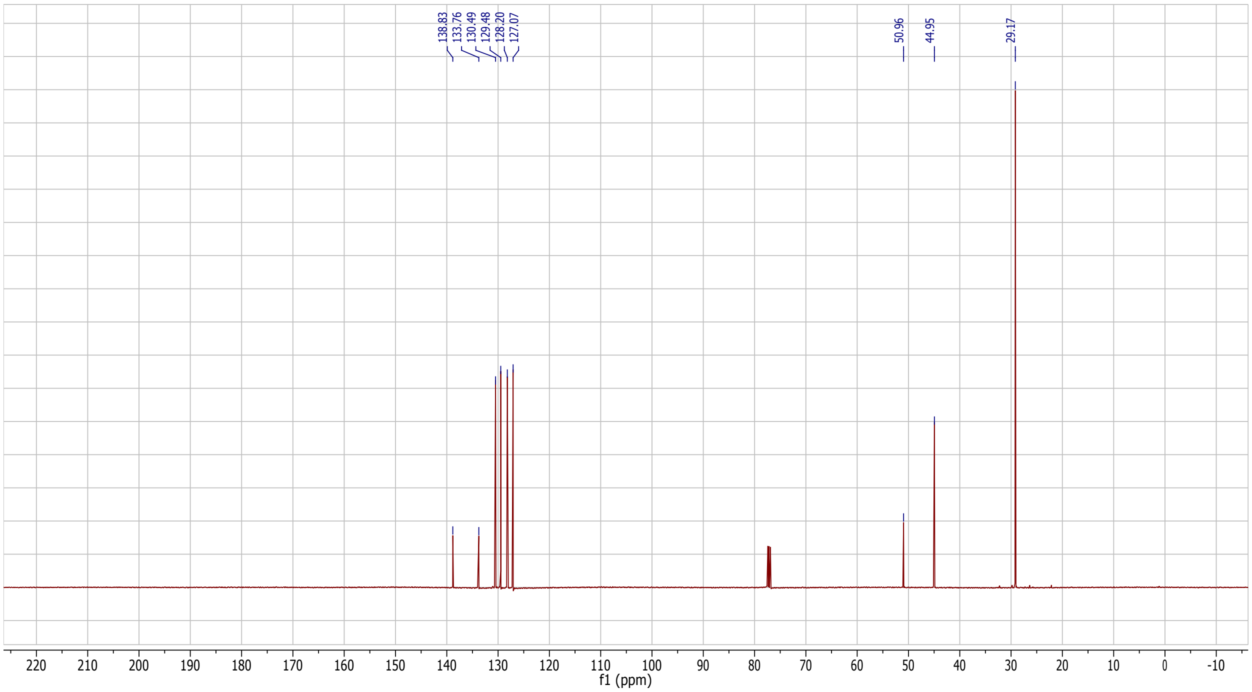

8-methyl-1,2,3,4-tetrahydroquinoline:[22]

Colourless oil, 64 mg, 87% yield. ^{1}H NMR ($CDCl_3$, 500 MHz, 298K): δ 6.96 (d, 1H, $^{3}J_{HH}$ = 7.6 Hz), 6.93 (d, 1H, $^{3}J_{HH}$ = 7.6 Hz), 6.64 (t, 1H, $^{3}J_{HH}$ = 7.6 Hz), 3.67 (s, 1H, br), 3.44 (t, 2H, $^{3}J_{HH}$ = 5.3 Hz), 2.87 (t, 2H, $^{3}J_{HH}$ = 6.6 Hz), 2.15 (s, 3H), 2.02 (m, 2H). ^{13}C NMR (126 MHz, $CDCl_3$, 298K): δ 142.8, 127.9, 127.4, 121.2, 120.9, 116.4, 42.4, 27.4, 22.2, 17.2. HRMS (DART-EI+): mass [M+H] calc'd for $^{12}C_{10}{}^{1}H_{14}{}^{14}N_1$: 148.11262, found: 148.11302.

^{1}H NMR ($CDCl_3$, 500 MHz, 298K):

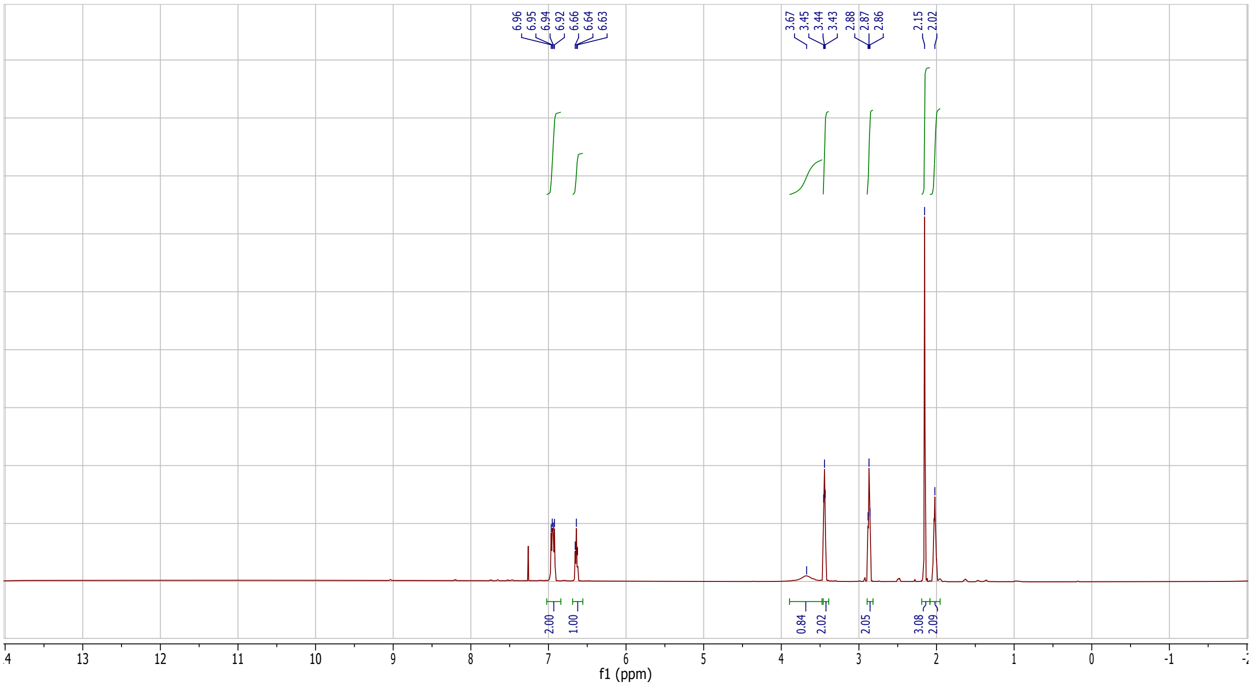

^{13}C NMR (126 MHz, $CDCl_3$, 298K):

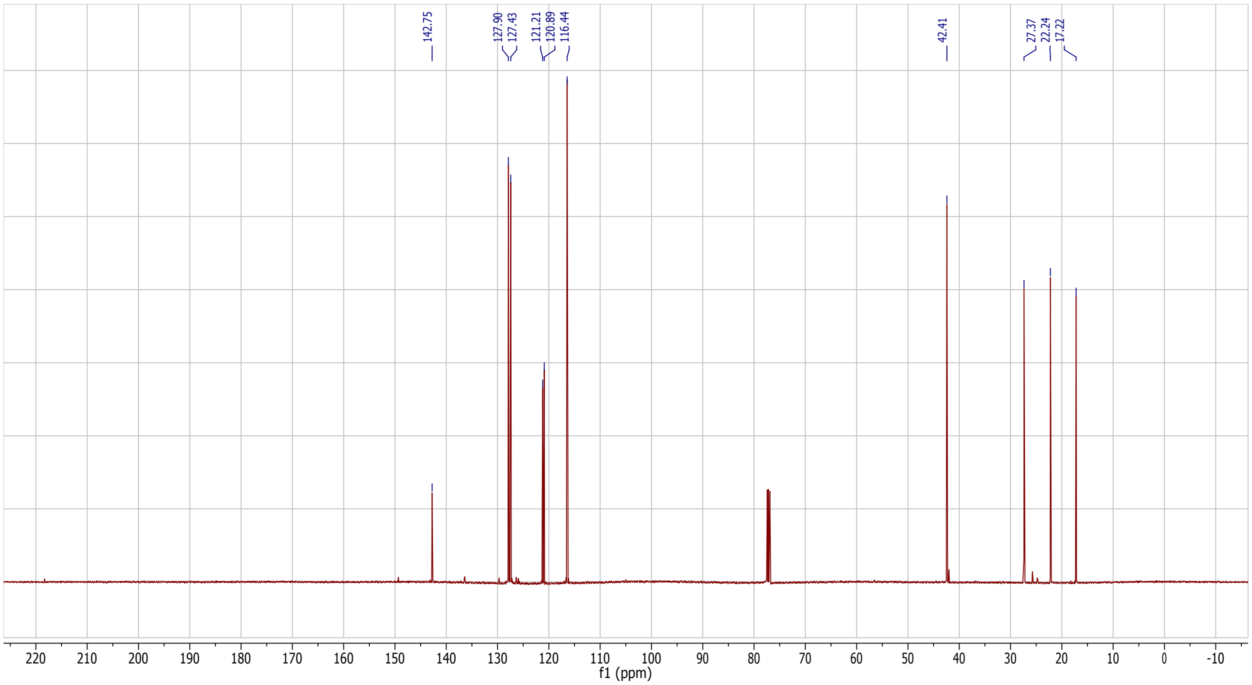

N-(1-(*p*-ethoxyphenyl)ethyl)aniline:[13]

Colourless oil, 115 mg, 95% yield. ^{1}H NMR ($CDCl_3$, 500 MHz, 298K): δ 7.34 (dm, 2H, $^{3}J_{HH}$ = 8.6 Hz), 7.17 (tm, 2H, $^{3}J_{HH}$ = 7.7 Hz), 6.92 (dm, 2H, $^{3}J_{HH}$ = 8.5 Hz), 6.72 (tm, 1H, $^{3}J_{HH}$ = 7.6 Hz), 6.60 (dm, 2H, $^{3}J_{HH}$ = 7.6 Hz), 4.51 (q, 1H, $^{3}J_{HH}$ = 6.7 Hz), 4.06 (q, 1H, $^{3}J_{HH}$ = 7.0 Hz), 1.55 (d, 3H, $^{3}J_{HH}$ = 6.7 Hz), 1.46 (d, 3H, $^{3}J_{HH}$ = 7.0 Hz), N-*H* not observed. ^{13}C NMR (126 MHz, $CDCl_3$, 298K): δ 157.9, 147.4, 137.2, 129.2, 127.0, 117.2, 114.6, 113.4, 63.4, 52.9, 25.0, 15.0. HRMS (DART-EI+): mass [M+H] calc'd for $^{12}C_{16}{}^{1}H_{20}{}^{14}N_{1}{}^{16}O_{1}$: 242.15449, found: 242.15366.

^1H NMR ($CDCl_3$, 500 MHz, 298K):

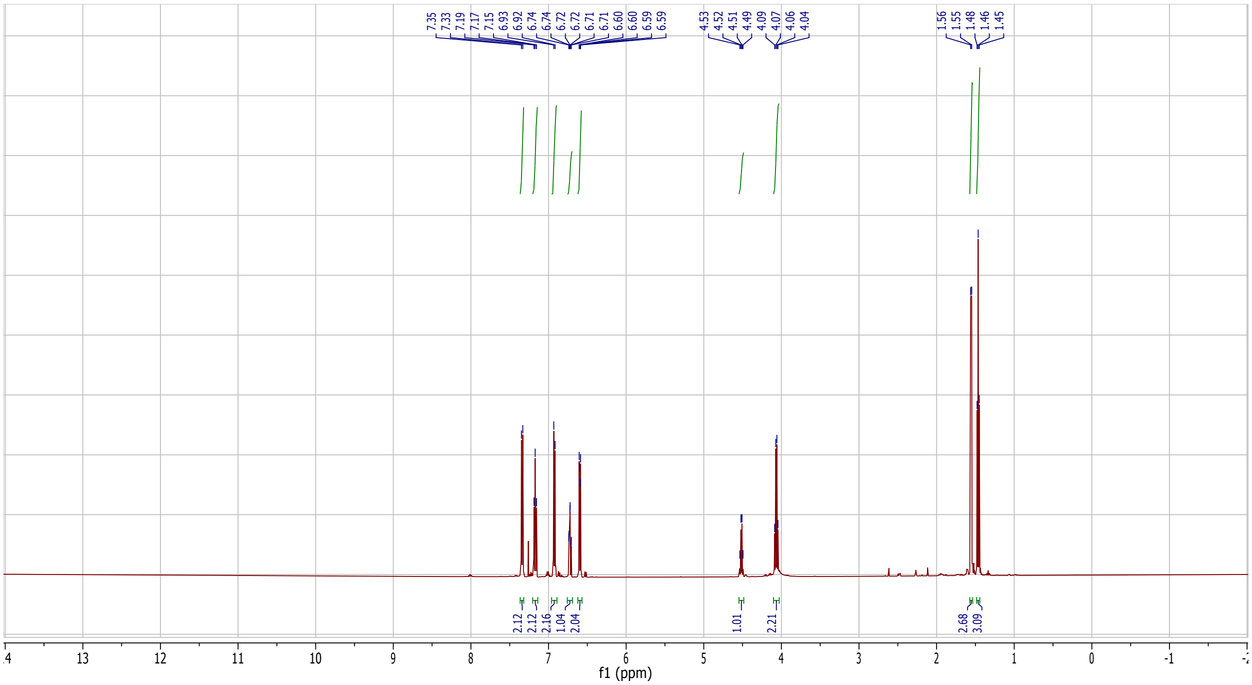

^{13}C NMR (126 MHz, $CDCl_3$, 298K):

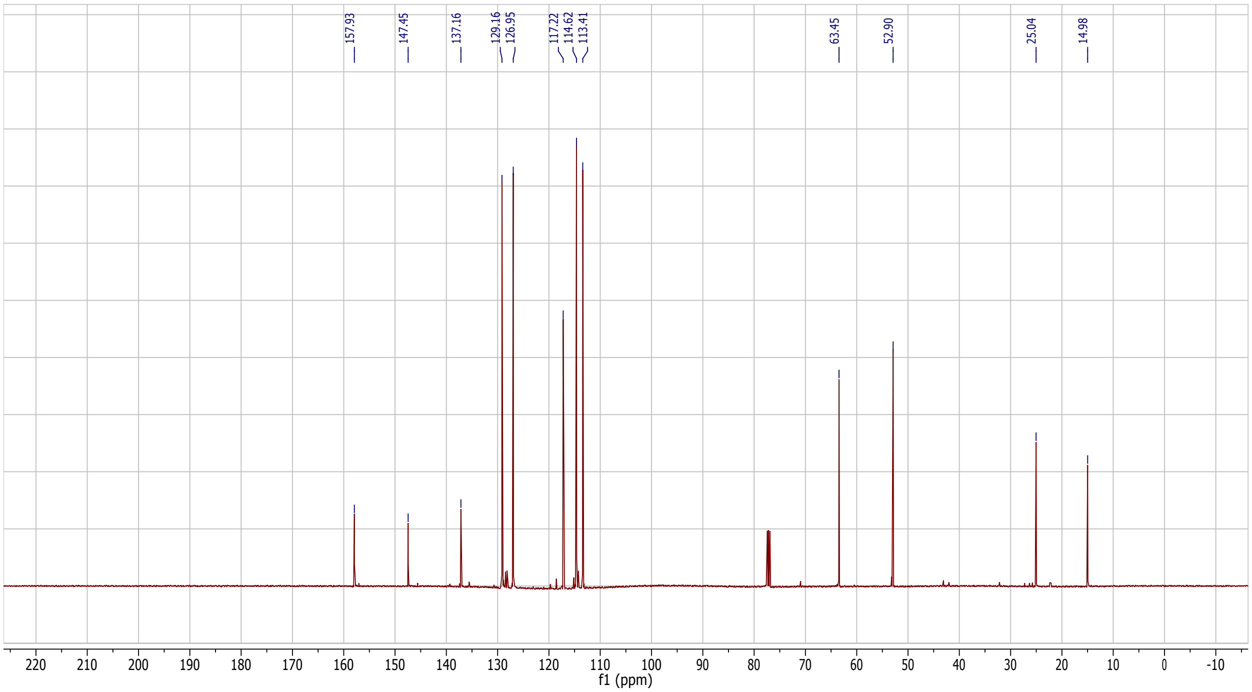

1,2,3,3-tetramethylindoline:[23]

Colourless oil, 71 mg, 81% yield. ^{1}H NMR ($CDCl_3$, 500 MHz, 298K): δ 7.16 (tm, 1H, $^3J_{HH}$ = 7.6 Hz), 7.09 (d, 1H, $^3J_{HH}$ = 7.2 Hz), 6.80 (t, 1H, $^3J_{HH}$ = 7.2 Hz), 6.58 (d, 1H, $^3J_{HH}$ = 7.8 Hz), 2.93 (q, 1H, $^3J_{HH}$ = 6.8 Hz), 2.76 (s, 3H), 1.35 (s, 3H), 1.25 (d, 3H, $^3J_{HH}$ = 6.6 Hz), 1.09 (s, 3H) N-*H* not observed. ^{13}C NMR (126 MHz, $CDCl_3$, 298K): δ 152.0, 139.4, 127.4, 121.7, 118.5, 107.8, 72.1, 42.8, 34.0, 25.8, 23.0, 12.3. HRMS (DART-EI+): mass [M+H] calc'd for $^{12}C_{12}{}^1H_{18}{}^{14}N_1$: 176.14392, found: 176.14451.

^{1}H NMR ($CDCl_3$, 500 MHz, 298K):

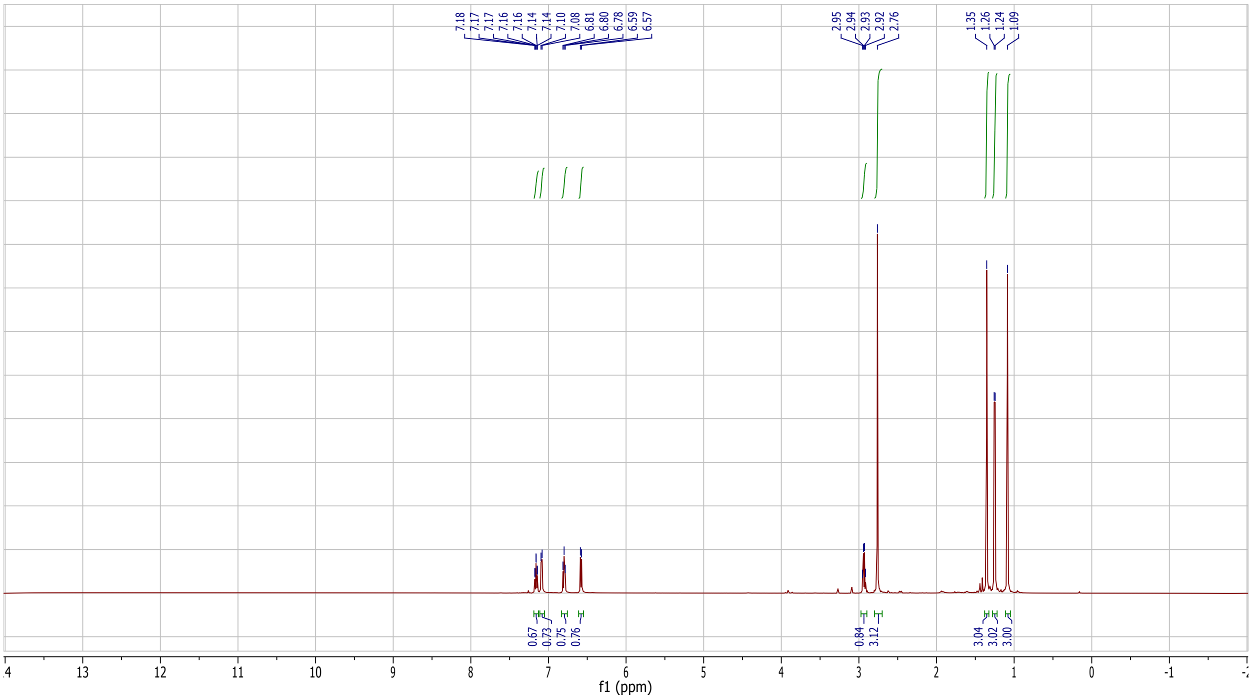

^{13}C NMR (126 MHz, $CDCl_3$, 298K):

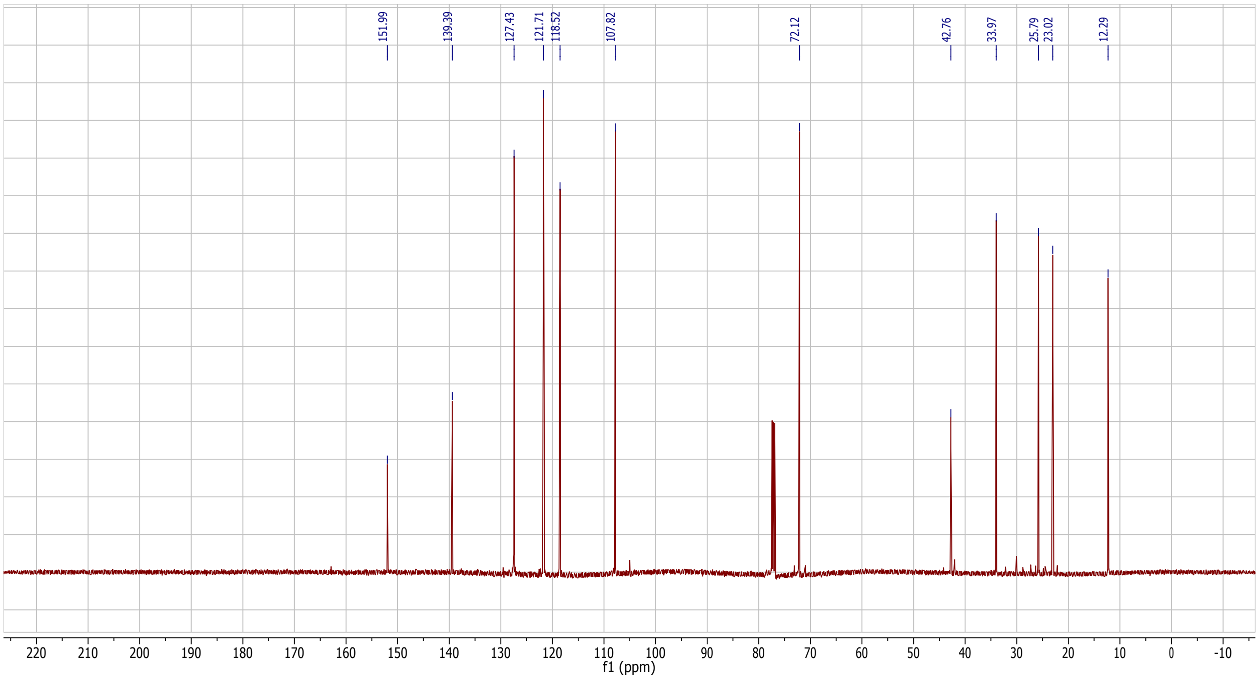

N-(1-phenylethyl)aniline:[24]

Colourless oil, 70 mg, 71% yield. ^{1}H NMR ($CDCl_3$, 500 MHz, 298K): δ 7.44-7.40 (m, 2H), 7.40-7.34 (m, 2H), 7.30-7.25 (m, 1H), 7.17-7.12 (m, 2H), 6.70 (tt, 1H, $^3J_{HH}$ = 7.2 Hz, $^4J_{HH}$ = 1.1 Hz), 6.59-6.55 (m, 2H), 4.54 (q, 1H, $^3J_{HH}$ = 6.7 Hz), 4.09 (s, br, 1H), 1.57 (d, 3H, $^3J_{HH}$ = 6.7 Hz) . ^{13}C NMR (126 MHz, $CDCl_3$, 298K): δ 147.4, 145.3, 129.2, 128.7, 127.0, 126.0, 117.4, 113.4, 53.58, 25.1. HRMS (DART-EI+): mass [M+H] calc'd for $^{12}C_{14}{}^{1}H_{16}{}^{14}N_1$: 198.12827, found: 198.12840.

^{1}H NMR ($CDCl_3$, 500 MHz, 298K):

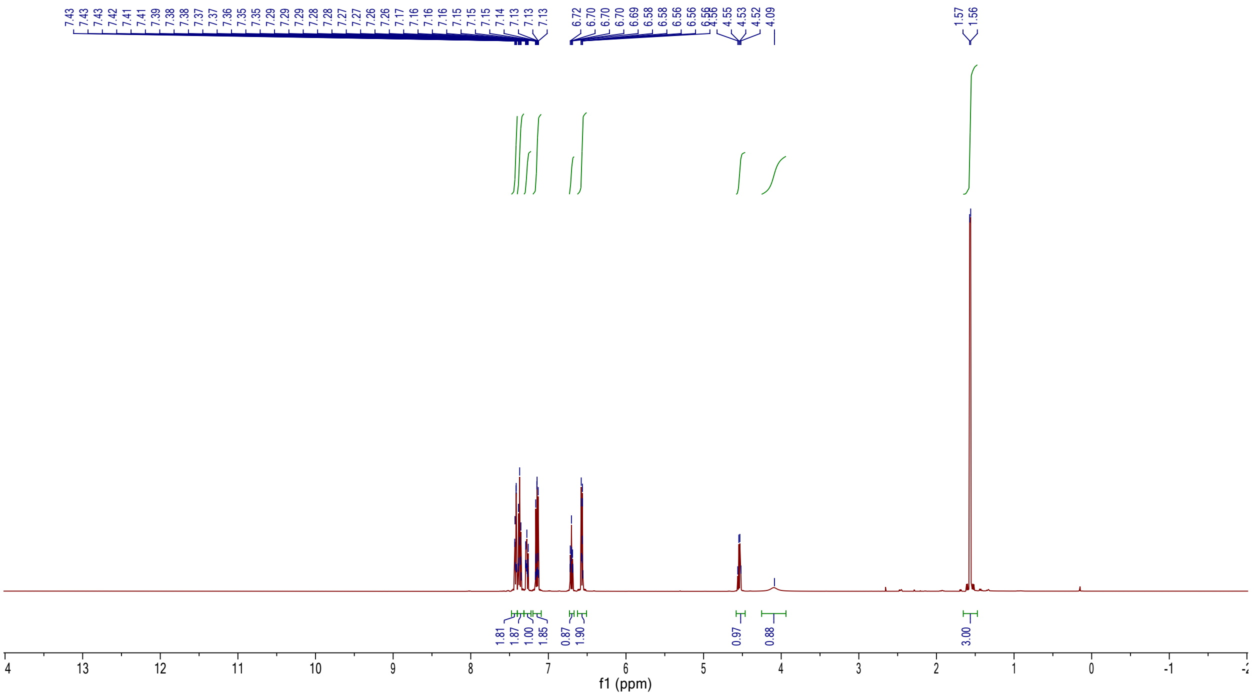

^{13}C NMR (126 MHz, $CDCl_3$, 298K):

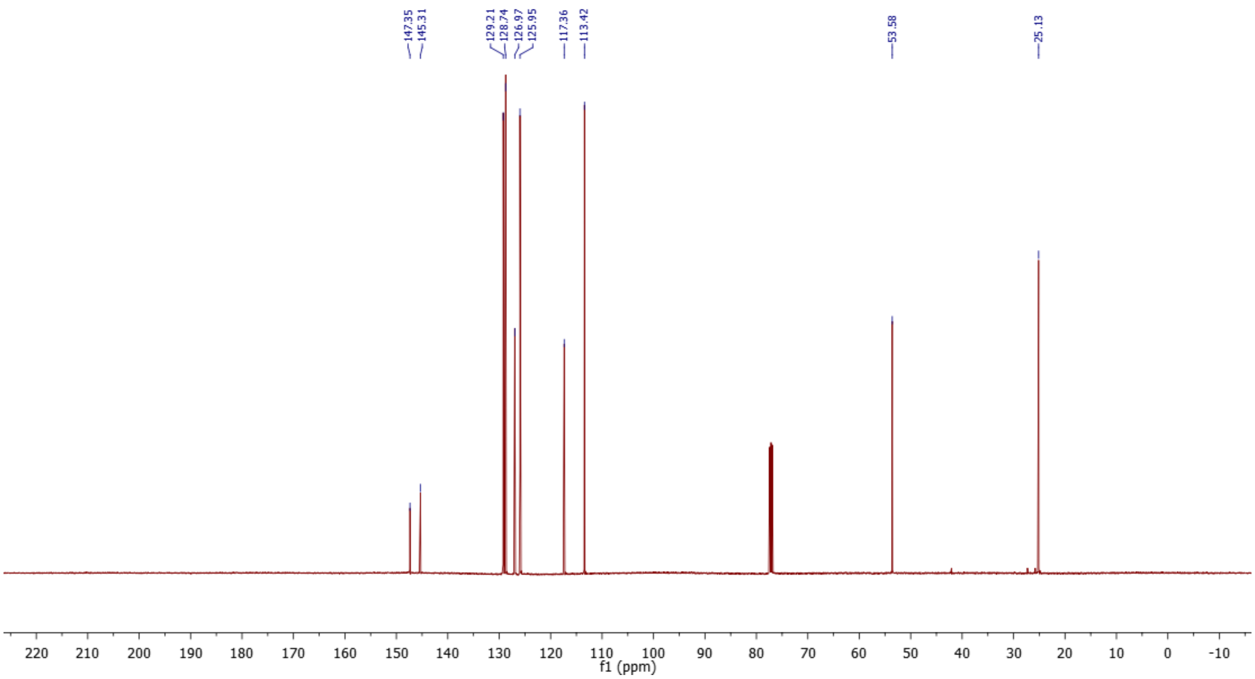

X-ray Crystallography

X-Ray Data Collection and Reduction

Crystals were coated in Paratone-N oil in the glovebox, mounted on a MiTegen Micromount and placed under an N_2 stream, thus maintaining a dry, O_2-free environment for each crystal. The data were collected on a Kappa Bruker Apex II diffractometer. Data collection strategies were determined using Bruker Apex 2 software. The data integration and absorption correction were performed with the Bruker Apex 2 software package. X-Ray Data Solution and Refinement Non-hydrogen atomic scattering factors were taken from the literature tabulations.[25] The heavy atom positions were determined using direct methods employing the SHELX-2013 direct methods routine. The remaining non-hydrogen atoms were located from successive difference.

Fourier map calculations.

The refinements were carried out by using full-matrix least squares techniques on F, minimizing the function $\omega\ (F_o\text{-}F_c)^2$ where the weight ω is defined as $4F_o^2/2\sigma\ (F_o^2)$ and F_o and F_c are the observed and calculated structure factor amplitudes, respectively. In the final cycles of each refinement, all non-hydrogen atoms were assigned anisotropic temperature factors in the absence of disorder or insufficient data. In the latter cases atoms were treated isotropically. C-H atom positions and H-atom temperature factors were calculated and allowed to ride on the carbon to which they are bonded assuming a C-H bond length of 0.95 Å. The H-atom contributions were calculated, but not refined. The locations of the largest peaks in the final difference Fourier map calculation as well as the magnitude of the residual electron densities in each case were of no chemical significance.

1. D. J. Parks, W. E. Piers and G. P. A. Yap, *Organometallics*, 1998, **17**, 5492-5503.

2. N. M. Scott, R. Dorta, E. D. Stevens, A. Correa, L. Cavallo and S. P. Nolan, *Journal of the American Chemical Society*, 2005, **127**, 3516-3526.

3. X. Bantreil and S. P. Nolan, *Nat. Protocols*, 2011, **6**, 69-77.

4. Y. Chu, H. Deng and J.-P. Cheng, *J. Org. Chem.*, 2007, **72**, 7790-7793.

5. C.-H. Hsieh, F.-I. Wu, C.-H. Fan, M.-J. Huang, K.-Y. Lu, P.-Y. Chou, Y.-H. O. Yang, S.-H. Wu, I. C. Chen, S.-H. Chou, K.-T. Wong and C.-H. Cheng, *Chem.--Eur. J.*, 2011, **17**, 9180-9187.

6. A. M. Oertel, V. Ritleng, L. Burr and M. J. Chetcuti, *Organometallics*, 2011, **30**, 6685-6691.

7. D. M. Khramov, V. M. Lynch and C. W. Bielawski, *Organometallics*, 2007, **26**, 6042-6049.

8. N. Kuhn, H. Bohnen, J. Kreutzberg, D. Blaser and R. Boese, *Chem. Commun.*, 1993, 1136-1137.

9. C. P. Casey and J. B. Johnson, *Journal of the American Chemical Society*, 2005, **127**, 1883-1894.

10. P. Franchi, C. Casati, E. Mezzina and M. Lucarini, *Organic & Biomolecular Chemistry*, 2011, **9**, 6396-6401.

11. J. N. Rosa, A. G. Santos and C. A. M. Afonso, *Journal of Molecular Catalysis A: Chemical*, 2004, **214**, 161-165.

12. M. C. Hansen and S. L. Buchwald, *Organic Letters*, 2000, **2**, 713-715.

13. Y. Liu and H. Du, *Journal of the American Chemical Society*, 2013, **135**, 6810-6813.

14. Y. Kuninobu, P. Yu and K. Takai, *Organic Letters*, 2010, **12**, 4274-4276.

15. H. C. Brown and S. U. Kulkarni, *Journal of Organometallic Chemistry*, 1979, **168**, 281-293.

16. D. A. Walker, T. J. Woodman, D. L. Hughes and M. Bochmann, *Organometallics*, 2001, **20**, 3772-3776.

17. D. M. Lindsay and D. McArthur, *Chemical Communications*, 2010, **46**, 2474-2476.

18. X. Pan, A. Boussonnière and D. P. Curran, *Journal of the American Chemical Society*, 2013, **135**, 14433-14437.

19. X. H. Yang, L. L. Zhao, T. Fox, Z. X. Wang and H. Berke, *Angewandte Chemie-International Edition*, 2010, **49**, 2058-2062.

20. P. Frøyen and P. Juvvik, *Tetrahedron Letters*, 1995, **36**, 9555-9558.

21. B. T. Cho and S. K. Kang, *Synlett*, 2004, **2004**, 1484-1488.

22. S.-I. Murahashi, Y. Imada and Y. Hirai, *Bulletin of the Chemical Society of Japan*, 1989, **62**, 2968-2976.

23. A. A. Tolmachev, *Khim. Geterotsikl. Soedin.*, 1986, **11**, 1474 -1477.

24. T. Kawakami, T. Sugimoto, I. Shibata, A. Baba, H. Matsuda and N. Sonoda, *The Journal of Organic Chemistry*, 1995, **60**, 2677-2682.

25. D. T. Cromer and J. T. Waber, *Int. Tables X-Ray Crystallogr.*, 1974, **4**, 71-147.
